# An in vitro study on factors affecting endotoxin neutralization in human plasma using the Limulus amebocyte lysate test

**DOI:** 10.1038/s41598-021-83487-4

**Published:** 2021-02-18

**Authors:** Stephan Harm, Claudia Schildböck, Karin Strobl, Jens Hartmann

**Affiliations:** Department for Biomedical Research, Danube University Krems, Krems, Austria

**Keywords:** Cytokines, Immunochemistry, Biochemistry, Immunology

## Abstract

Endotoxin neutralization, caused by plasma components, makes it difficult to detect endotoxins in human blood. In this study, we investigated which factors influence the recovery of endotoxins using limulus ameobocyte lysate (LAL)-based assays. The individual factors that were examined in more detail were lipoprotein content, type of blood anticoagulation, kinetics and serum levels of divalent cations. Furthermore, it was investigated whether there is a direct correlation between LAL activity and monocyte activation. We could show that polyanionic heparin increases endotoxin recovery in blood, while citrate anticoagulation promotes endotoxin neutralization. Furthermore, we could show that the endotoxin activity in human plasma and serum decreases strongly over time. Time-dependent endotoxin neutralization reaches its maximum after 4–6 h incubation. By means of filtration tests we could determine that endotoxins in the plasma bind to lipoproteins but do not influence their activity. Comparative measurements have shown that high LAL activity of endotoxins in plasma simultaneously possesses high monocyte activating properties in whole blood. For the maximum recovery of endotoxins in human blood the physiological calcium and magnesium concentrations are sufficient. In this study, it was shown that the endotoxin neutralizing plasma components have a molecular weight similar to β2-microglobulin (11.7 kDa). For the exact identification of the endotoxin neutralizing plasma components, which caused a modulation of the immunostimulating endotoxin activity, further investigations have to be carried out in the future.

## Introduction

**Figure 1:**
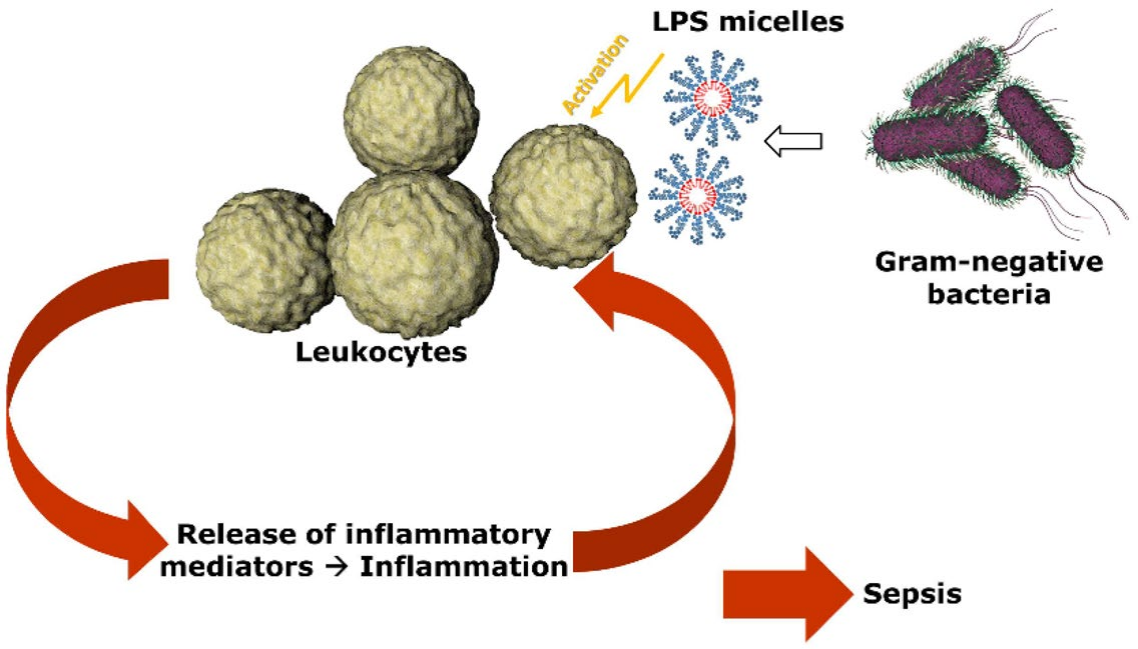
LPS stimulate the innate immunity. LPS in the blood activate monocytes, which leads to the secretion of inflammatory mediators. In systemic inflammation, this can lead to multiorgan failure and sepsis.

Endotoxin is a major constituent of the outer cell wall of Gram-negative bacteria and is highly toxic. Intravenous doses as low as 1 ng/kg body weight per hour cause an inflammatory response in humans^1^. Endotoxins are lipopolysaccharides (LPS), which are composed of a hydrophilic polysaccharide and a lipophilic lipid domain (lipid A moiety). LPS is a strong stimulator of inflammatory reactions, acts at very low concentrations, and is involved in the pathogenesis of sepsis, septic shock and endotoxemia^2^. LPS is released from the cell wall of growing bacteria or when antibiotics or the complement system kill bacteria^3^. LPS that enters the circulatory system initiates the inflammatory response of monocytes and macrophages which leads to the production of pro-inflammatory cytokines such as TNF-α and IL-1β^4^, and activates the production of co-stimulatory molecules required for the adaptive immune response^5^. However, in severe cases, the inflammation gets out of control and can finally result in sepsis or organ failure (Fig. 1).

**Figure 2:**
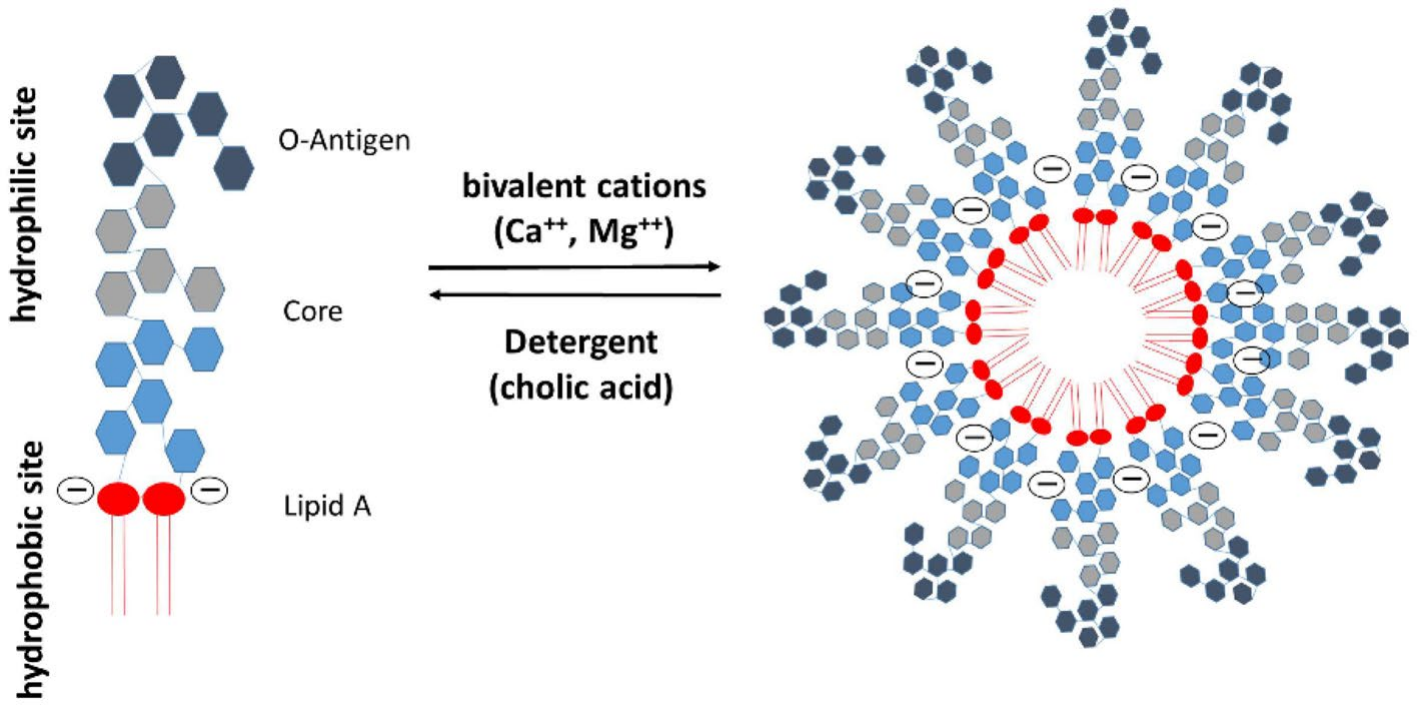
Structure of LPS aggregates. In aqueous solution monomecric LPS molecules form aggregates in the form of micelles or vesicles. Bivalent cations form bridges between the negatively charged phosphate groups in the lipid A fraction. By adding chelators (EDTA, citrate) and detergents (DOC, Tween, …), the LPS aggregates can be destabilized and disintegrate into monomers. In contrast to LPS aggregates, LPS monomers are no longer active both in the LAL cascade and immunologically (monocyte activation)^14,15^.

If a sensitive and reliable test for the detection of LPS in the blood were available, it would be possible to start a targeted antibiotic therapy against Gram-negative pathogens very early. The Limulus Amebocyte Lysate (LAL) test is the most established method for quantifying LPS. The LAL test measures the coagulation of a lysate derived from blood cells (amebocytes) of the horseshoe crab (*Limulus polyphemus*) caused by endotoxin. After activation of factors C and B by lipopolysaccharide, a coagulation enzyme activates coagulation, which is then evaluated turbidimetrically or by means of a color reaction^6^. The LAL test is used successfully for detection of LPS in a wide variety of industrial, pharmaceutical, and research samples, including water supplies, parenteral fluids, drugs for intravenous administration, and certain biologic fluids (e.g., cerebrospinal fluid). In contrast to the widespread use of the LAL test in industrial pharmaceutical settings, the application of the LAL test for the detection of LPS in blood has been significantly hindered by the presence of inhibitors^7^. To overcome these inhibitions, several approaches of plasma treatment prior to the LAL test have been described. Of these methods, the most widespread experience has been obtained with dilution plus heating^8^. By diluting the samples, LAL inhibiting substances are less interfering while heating denatures plamatic serine proteases, which influence the LAL measurement^8,9^. LPS typically consists of a hydrophobic domain known as lipid A, a nonrepeating “core” oligosaccharide and a distal polysaccharide (O-antigen). As an amphiphilic molecule, LPS is known to form aggregates with high molecular masses of more than 10^6^ Da in hydrophilic environments. The size of these aggregates depends on parameters such as temperature, pH, hydrophilicity and the presence of mono- or divalent cations^10^. LPS inserts into the aggregate via integration of the acyl chains into the aggregate interior, with the polysaccharide portions are fully exposed to the aqueous compartment^10^ (Fig. 2). The question whether monomeric LPS molecules or large endotoxin aggregates are responsible for the activation of the immune system is controversially answered in the literature. Takayama et al.^11,12^ found that LPS monomers exhibit significantly higher activity both in the LAL cascade and in the activation of a pro-B cell line. Many other studies indicate that LPS aggregates are responsible for the activation of the LPS cascade as well as for the activation of the immune system^13–15^. The geometric form of LPS aggregates depends strongly on the chemical structure of the aggregating molecules. Studies on the role of the aggregate structure for biological activity have shown that cubic LPS aggregate structures have a high endotoxic activity, while lamellar aggregate structures have little or no endotoxic activity^16^. This dependence has been demonstrated for a variety of endotoxin and non-endotoxin structures^17^. Although this fact has been known for many years, there is only little information about LPS aggregate structure and aggregate size in human plasma and blood^18^.

The LAL activity of LPS is completely lost in 0.5% sodium deoxycholate (DOC), but is recovered after DOC is reduced by dilution or dialysis. This indicates that a minimum size of LPS is necessary to induce pyrogenicity. Ribi et al.^15^ reported the effect of human plasma on the LAL activity of LPS in DOC. They demonstrated that LPS potency in DOC was recovered after dilution. However, the potency was not recovered by dilution if human plasma was added, and direct injection of LPS in DOC did not show any LAL activity. These results suggested that human plasma prevented the reassembly of LPS by dilution. A possible explanation for the neutralization of endotoxins in human blood is described by the endotoxin-lipoprotein hypothesis, established by Rauchhaus et al.^19^. The hypothesis is that LPS binds to lipoproteins (LDL, HDL, VDL, lipoprotein(a), triglycerides, chylomicrons) and thus their biological activity is reduced. In 1992 C. Weinstock et al. found that the biological activity of LPS is reduced by LDL^20^. In vitro and in vivo experiments have suggested that lipoproteins can neutralize LPS and have an immune-modulating effect on macrophages as well as an anti-inflammatory effect when endothelial cells are activated^21–25^. In summary, LPS bound to lipoproteins does not bind to macrophages and is 100- to 1000-fold less active than free LPS regarding monocyte activation^26^. In addition, a faster clearance of LPS through the liver was observed by chylomicron administration^27^. This effect is also known as the liver's immunomodulatory response to infections. The combination of positively charged apolipoprotein B and the hydrophobic phospholipid layer of the lipoprotein vesicle cause the binding of LPS to lipoproteins^28^.

That coagulation of blood samples greatly reduces endotoxin recovery was published by Jevin et al.^7^. Armstrong et al.^29^ found that both, cellular and plasmatic coagulation in mammals entrap endotoxins in blood and affect endotoxin levels in blood samples. As a result, serum samples should not be used for clinical endotoxin testing.

**Figure 3:**
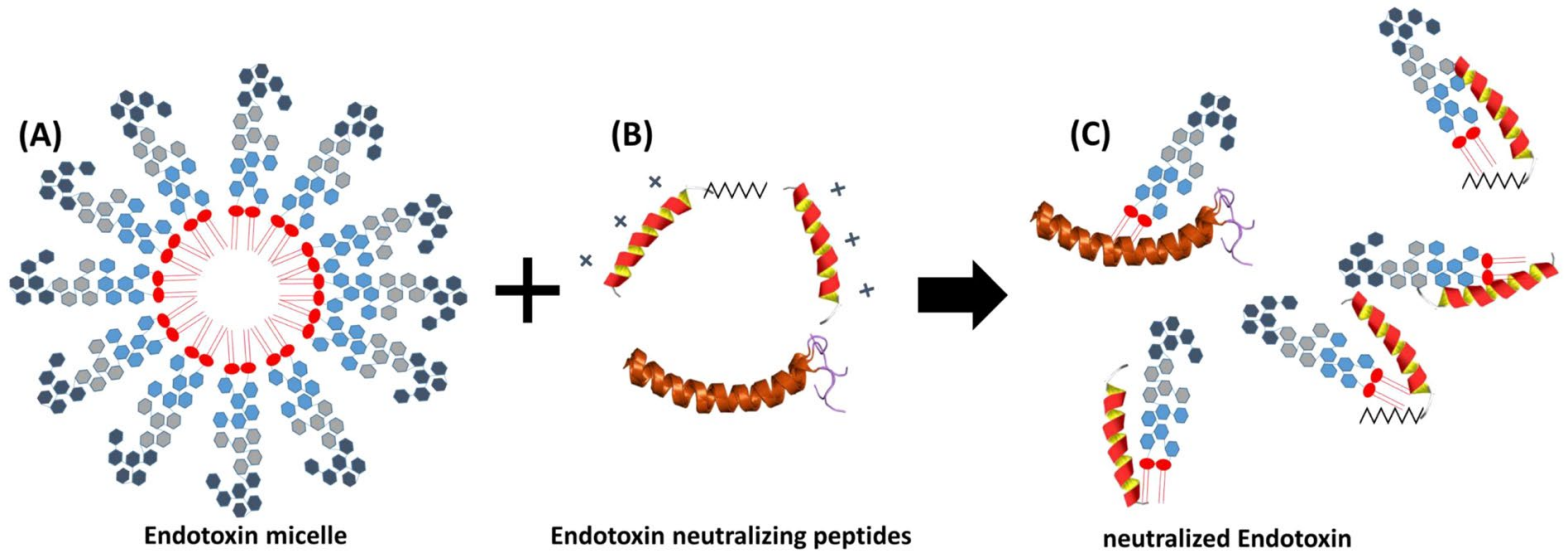
Endotoxin neutralization caused by antimicrobial peptides. Endotoxin monomers form micelles or aggregates with a size between 100 and 1000 nm (**A**). Cationic antimicrobial peptides and proteins (**B**), which are part of the innate immune system, bind the endotoxin monomers and neutralize their inflammatory effect (**C**). The neutralized LPS are no longer detectable in the LAL test.

For the sake of completeness, it should be noted that there are cationic plasma proteins such as bactericidal permeability increasing protein (BPI) and lactoferrin that can bind LPS and inhibit endotoxin activity. These endotoxin binding proteins are secreted by granules and neutrophils during inflammatory responses. A number of studies have shown that antimicrobial peptides (AMPs) also have the potential to neutralize LPS-induced endotoxic effects (Fig. 3). These peptides are important components of the innate immunity^30^. They are produced at the site of infection and/or inflammation and act rapidly to clear microbes^31^. Several AMPs prevent LPS-dependent cytokine induction in macrophages and block sepsis in animal models^32–35^. These studies also showed a direct correlation between the ability of AMPs to bind to LPS and their antimicrobial activity. AMPs display a high content of cationic and hydrophobic amino acids. It is widely believed that they act through nonspecific binding to biological membranes, even though the exact nature of these interactions is currently unclear^36,37^. Because AMPs are cationic, binding to the polyanionic heparin is obvious^38^.

In the literature, it is pointed out that LPS activity in plasma also depends on the anticoagulant^39,40^. It was observed that the LPS activity in heparinized plasma is significantly higher than in plasma anticoagulated with EDTA or citrate. The explanation of this finding was the reduction of the ionic calcium and magnesium levels, which are responsible for the stability of the LPS micelles. Reich et al. found that endotoxin neutralization caused by citrate or other complexing agents is not reversible^41^. Endotoxin determination in a sample is considered valid, if 50–200% of the spiked endotoxin is recovered. The percentage recovery is based on the LAL value found in depyrogenated water with the same amount of endotoxin^41–43^. When the LAL test is used to detect endotoxin in blood, we encounter two obstacles in addition to those described for fluids other than blood. First, the complex and poorly understood inhibitory factors present in blood and second, the degree of endotoxemia, which is generally at the detection limit of the test^44^. Endotoxin interacts with several components of the plasma, including bile salts, proteins and lipoproteins, resulting in disaggregation, some inactivation and complex formation. A reliable method for endotoxin quantification in human plasma is therefore not available with the LAL test^44^. It should be noted that the LAL test has not yet been approved by the Food and Drug Administration for the detection of endotoxemia in clinical practice, and the currently licensed kits indicate that they are not approved for this purpose.

In the present study we compared the LAL activity between LPS spiked serum and different anticoagulated human plasma spiked with LPS using the chromogenic LAL assay from Charles River (Wilmington, USA). Additionally we tested the endotoxin activity in human plasma as a function of divalent cation content (Ca^2+^, Mg^2+^). In the course of this study, we also checked if the endotoxin activity correlates with the lipoprotein content in human plasma. Furthermore, it was examined whether the LAL activity correlates with monocyte activation in LPS-spiked human blood. The time-dependency of endotoxin activity with respect to LAL activity and monocyte activation was also examined more closely. Finally, heparin-binding plasma components were enriched using a heparin-immobilized adsorbent to check their ability to mask endotoxins. The aim of this study was to identify the LAL inhibiting factors in blood in order to establish a sample procedure that would allow a standardized quantification of endotoxins in human blood.

## Results

### Endotoxin activity in human serum/plasma and influence of anticoagulation

**Figure 4:**
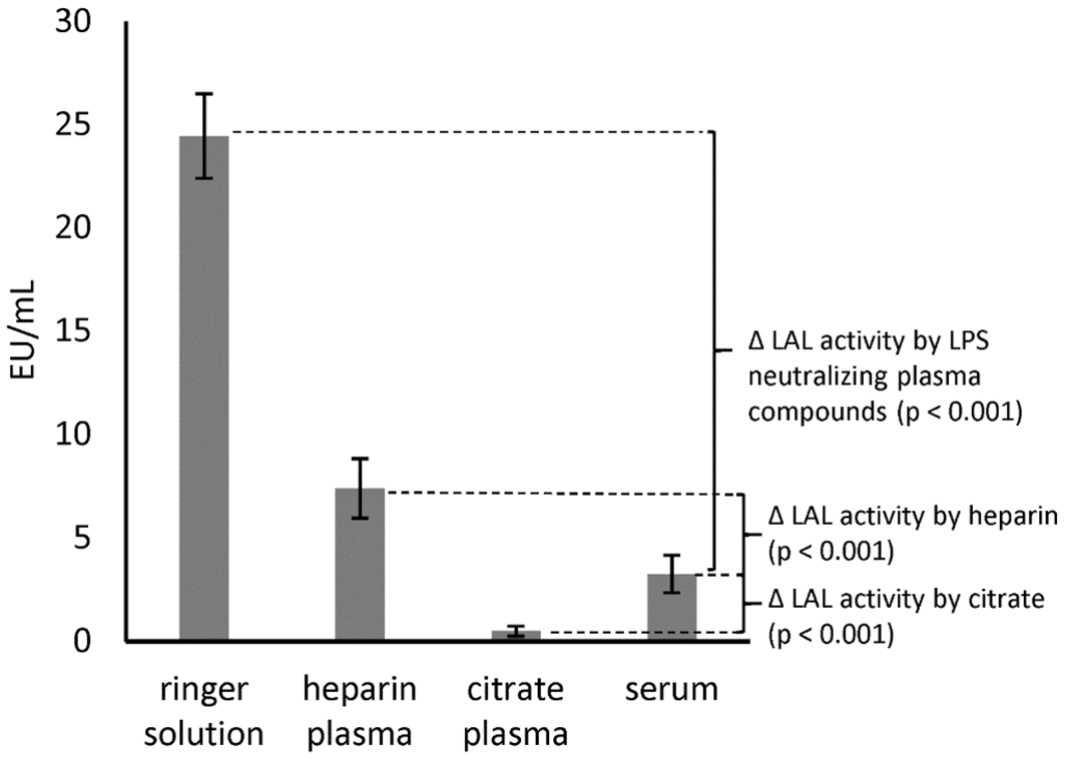
Comparison of LPS activity in aqueous solution, serum, heparin plasma and citrate plasma. 5 ng/mL LPS (*E. coli*) was spiked into ringer solution, human serum, heparin plasma and citrate plasma**.** The recovery of the added LPS into the different medium was determined by the LAL test. Mean ± standard deviation of three independent experiments with plasma/serum from different donors are shown.

In this study, we first compared the endotoxin activity of 5 ng/mL LPS (*E. coli*) in aqueous solution (ringer solution) with human serum, citrated (17 mM) plasma and heparin (17 IU/mL) plasma (Fig. 4).

In human serum, the LAL recovery of 5 ng/mL LPS (*E. coli*) was only 13 ± 4% compared to ringer solution. In citrated plasma, the LAL recovery was even lower, namely 2 ± 1%. Divalent cations serve as an ion bridge between the anionic phosphate groups in the lipid A portion of the LPS molecule and contribute significantly to the stability of the LPS aggregates. The destabilization of LPS aggregates through the removal of divalent ions by means of citrate thus leads to an increased neutralization of endotoxins in human plasma. Surprisingly, the recovery of LPS in heparin plasma was with 30 ± 6% significantly higher than in serum (Fig. 4). This means that the endotoxin activity in heparin plasma was 138 ± 51% higher than in serum. A possible explanation could be that polyanionic heparin binds and deactivates positively charged proteins responsible for endotoxin neutralization.

**Figure 5:**
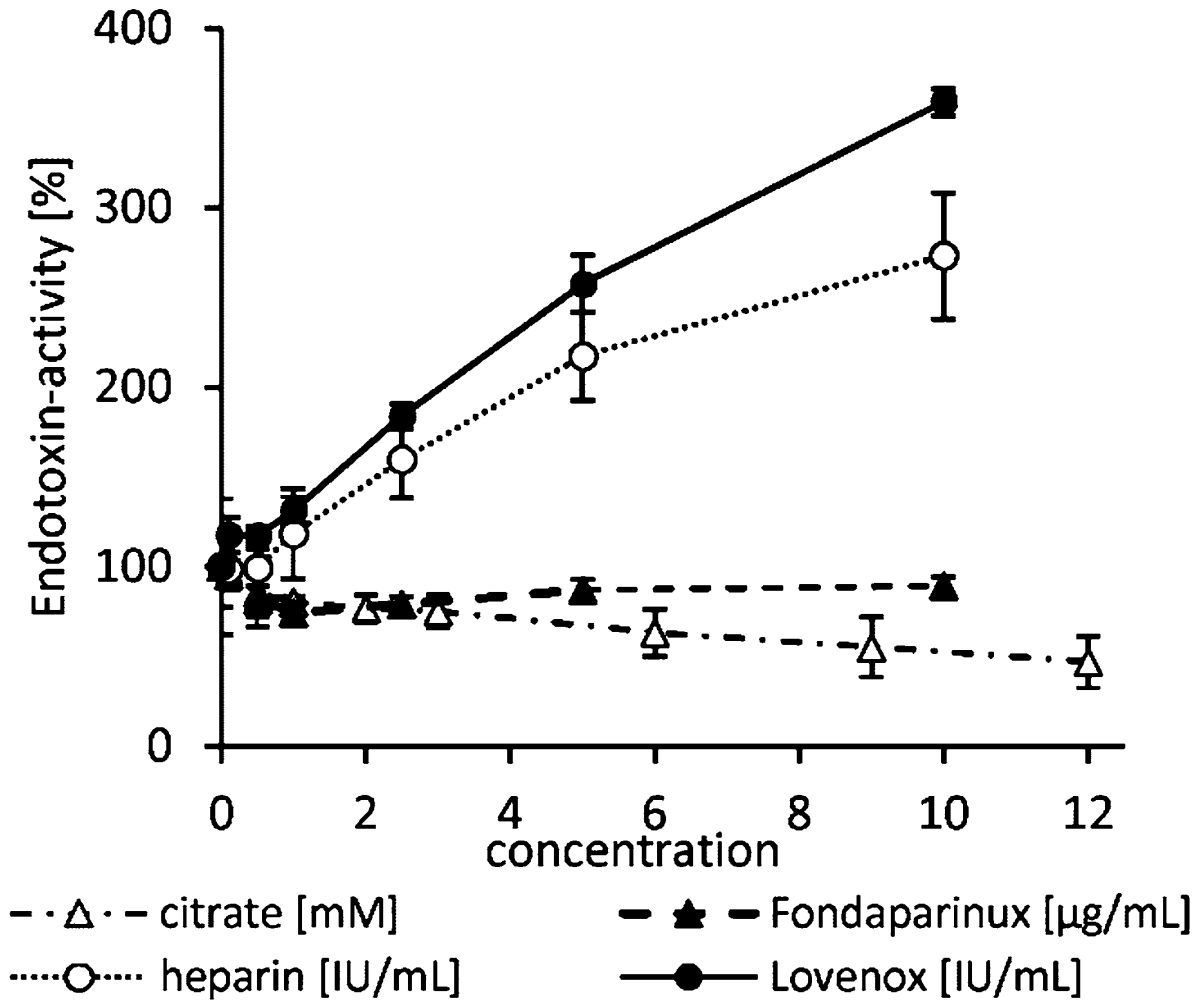
Influence of different anticoagulants on endotoxin activity in plasma. Increasing concentrations of unfractionated heparin, lovenox, fondaparinux and citrate was added to human serum. Subsequently, 5 ng/mL LPS (*E. coli*) was spiked and the endotoxin activity was measured by LAL test. The endotoxin activity is expressed as a percentage and refers to the serum sample without anticoagulant set at 100%. Mean ± standard deviation of three independent experiments with plasma/serum from different donors are shown.

As we found a significantly different LPS recovery in serum compared to anticoagulated plasma, the next step was to test the endotoxin activity depending on the concentration of selected anticoagulants. The anticoagulants tested in human plasma were unfractionated heparin, low molecular weight heparin (Lovenox) and fondaparinux (activated factor X inhibitor) in the concentration range between 0 to 10 IU/mL. Citrate dependent endotoxin activity in plasma was tested up to 12 mM (Fig. 5). Fondaparinux shows no effect on the LPS activity in the LAL test at the tested concentrations. Using 2.5 IU/mL and higher serum levels of unfractionated or low-molecular-weight heparin as anticoagulants, significantly (p ≤ 0.05) higher endotoxin values were obtained by the LAL test. The endotoxin activity increased continuously by increasing heparin additions up to the maximum tested concentration of 10 IU/mL where the endotoxin activity is 259 ± 8% higher in case of unfractionated heparin and 173 ± 35% higher when fractionated heparin (Lovenox) is used compared to the LAL measurement of 5 ng/mL LPS in serum without anticoagulant. With the addition of citrate, the endotoxin activity decreases. From a citrate serum level of 3 mM on, a significant (p ≤ 0.05) reduction of endotoxin activity was measured.

**Figure 6:**
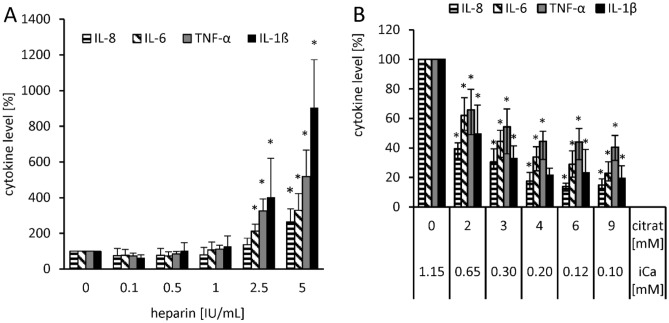
Influence of heparin and citrate dose on the stimulating property of endotoxin on monocytes. Serum with increasing concentrations of heparin (**A**) or citrate (**B**) was mixed with fresh blood cells in a 1:1 ratio (volume serum:volume blood cells), spiked with 10 ng/mL LPS and then incubated at 37 °C for 4 h. Activation of TLR4 of monocytes was determined by cytokine release. Mean ± standard deviation of three independent experiments with blood from three different donors are shown. The bars marked with * show a significantly (p ≤ 0.05) higher (**A**) or lower (**B**) cytokine level compared to sample without anticoagulants.

In order to determine whether heparin and citrate actually change endotoxin activity or whether heparin influences the LAL clotting cascade and thus the analysis, the LPS-induced cytokine release of monocytes was measured in a separate experiment. Fresh blood with increasing heparin and citrate concentrations was spiked with LPS and after an incubation period of 4 h the cytokines (IL-1β, IL-6, IL-8 and TNF-α), which were secreted by LPS stimulated monocytes, were measured. When 2.5 IU/mL heparin was used, a significant (p ≤ 0.05) increase in secreted cytokines could be measured, except for IL-8. At a serum level of 5 IU/mL, the measured cytokine release was between 164 ± 73 and 805 ± 268% higher than in non-heparinized blood (Fig. 6A). When citrate was used as anticoagulant in this experiment, a significant (p ≤ 0.05) reduction of LPS induced cytokine release could already be measured at a serum level of 2 mM (Fig. 6B, Table 1). At a citrate level of 9 mM the cytokine levels decreased to 15 ± 4 to 41 ± 9% compared to non-anticoagulated serum.

**Table 1:** Cytokine levels of LPS spiked whole blood as a function of citrate level. Fresh blood from three different donors was spiked with different amounts of citrate. After addition of 10 ng/mL LPS and 4 h incubation at 37 °C, the cytokine serum levels were determined.

Citrate (mM)	Cytokine serum level (pg/mL)			
	IL-1β	IL-6	IL-8	TNF-α
0	844 ± 341	13,829 ± 4437	3423 ± 1119	8707 ± 1342
2	375 ± 93	8332 ± 1688	1402 ± 628	5702 ± 1573
3	257 ± 88	5892 ± 587	1085 ± 561	4629 ± 1132
4	170 ± 68	4456 ± 439	641 ± 363	3805 ± 802
6	164 ± 12	3799 ± 657	474 ± 192	3803 ± 1057
9	145 ± 47	3054 ± 714	538 ± 287	3519 ± 907

### Influence of calcium and magnesium serum levels on endotoxin activity

**Figure 7:**
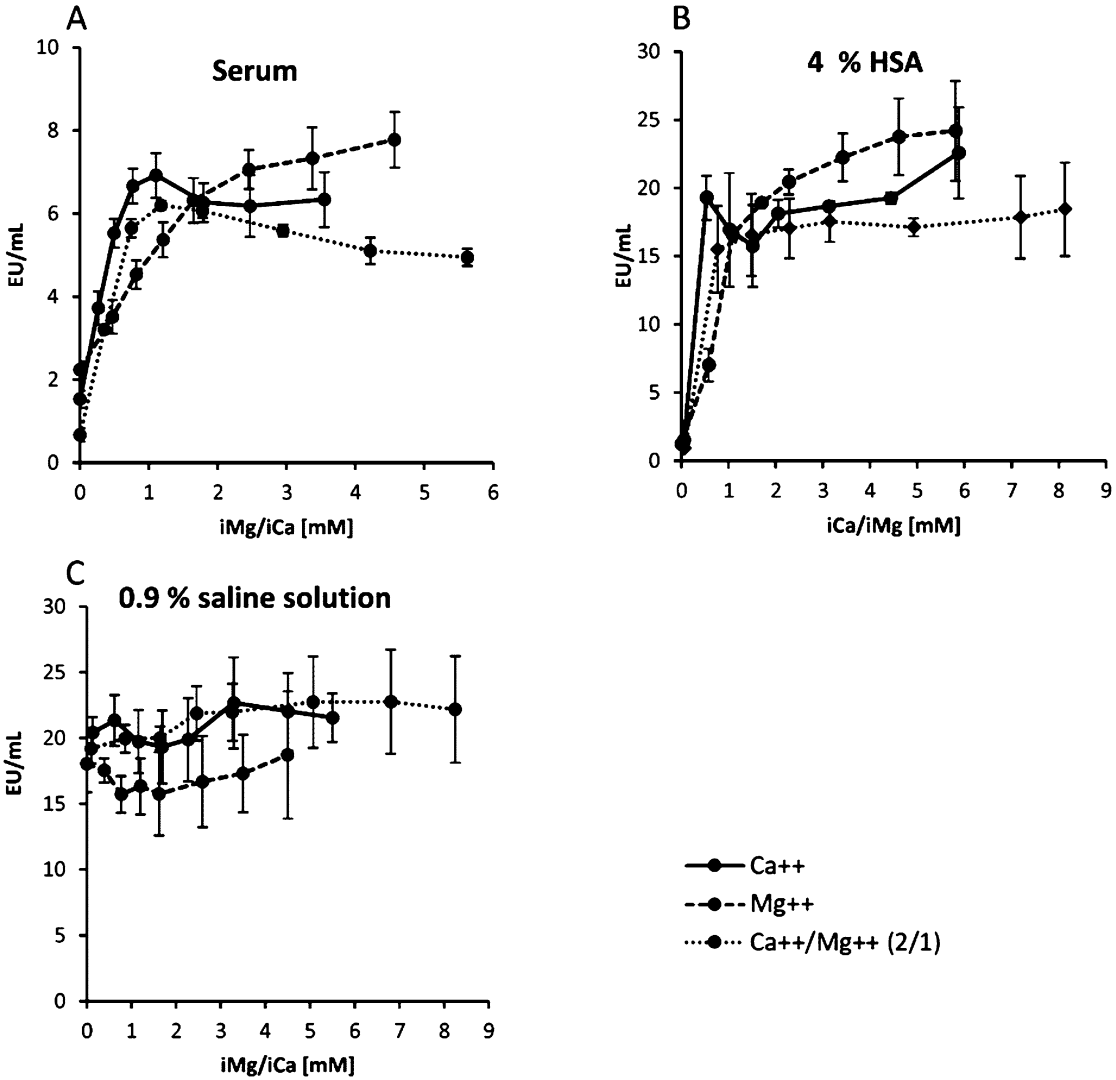
Endotoxin activity in serum (**A**), 4% HSA solution (**B**) and aqueous protein-free saline solution (**C**) as a function of divalent cation levels. The three media were spiked with increasing concentrations of divalent cations (iCa, iMg or iCa/iMg in 2:1 ratio). The x-axis shows the sum of iCa + iMg value in mM. Afterwards, the recovery of 5 ng/mL LPS (*E. coli*) was determined by LAL test. Mean ± standard deviation of three independent experiments are shown.

As a next step, the endotoxin activity of 5 ng/mL LPS was systematically tested as a function of divalent cation levels. The ion-dependent LAL activity of endotoxins was compared between serum, 4% human albumin solution and protein-free physiological saline solution. Calcium and magnesium-free serum was prepared by pretreatment with a cation exchanger. Calcium and magnesium-free serum, HSA solution and the saline solution were spiked with increasing concentrations of calcium chloride, magnesium chloride and a mixture of calcium and magnesium chloride solution in physiological ratio of 2:1. Subsequently, the samples were spiked with 5 ng/mL LPS (*E. coli*) and their endotoxin recovery was determined by LAL test. The ion concentrations were monitored with an electrolyte analyzer. In serum and in the 4% HSA solution a significant (p ≤ 0.05) increase in endotoxin activity can be measured in the LAL test even at the lowest dosage (0.5 mM) of CaCl_2_, MgCl_2_ or CaCl_2_/MgCl_2_ solution (Fig. 7). In serum the maximum LAL recovery is achieved at an ionized calcium level (iCa) of 1.1 ± 0.04 mM (Fig. 7A). Higher concentrations of iCa in serum cause a slight decrease in the measured LAL values. In case of ionized magnesium (iMg) a significant (p ≤ 0.05) increase of the measured endotoxin activity up to 2.5 ± 0.11 mM is observed. With further increase of the iMg level the LAL measurement is only slightly increased. Intensive research in the past to optimize LAL measurements has shown that magnesium is a far more important cofactor in the LAL cascade than calcium. This is probably due to the fact that the Atlantic horseshoe crab, from which the LAL reagent is derived, developed in an environment (seawater) where the iMg:iCa ratio is 50:10^45^. When adding a mixture of calcium and magnesium chloride (iCa/iMg) in physiological 2:1 molar ratio, the maximum endotoxin value is already achieved at 0.72 ± 0.06 mM iCa and 0.45 ± 0.05 mM iMg. At higher dosages, a decrease of the LPS recovery can be observed. With the addition of calcium and magnesium as well as with the single addition of calcium only, it can be observed that above the physiological value the recovery of endotoxin in serum decreases (Fig. 7A). Since calcium serves as a cofactor for many biochemical processes in the blood, the calcium level in human blood is highly regulated and controlled. The smallest deviations cause the activation of various enzymes such as serine proteases in blood coagulation. Possibly, changes in enzyme activities in serum also affect LAL recovery. To confirm this assumption, however, separate experiments would have to be performed.

In 4% HSA solution, maximum LPS recovery is achieved even at the lowest tested iCa concentration (0.55 ± 0.05 mM) (Fig. 7B). An increase in the iCa serum level up to the maximum tested iCa concentration of 5.88 ± 0.03 mM does not cause a change of the LAL values. The addition of divalent cations to protein-free, physiological saline solution does not affect LPS recovery (Fig. 7C).

### Endotoxin activity in serum as a function of incubation time

**Figure 8:**
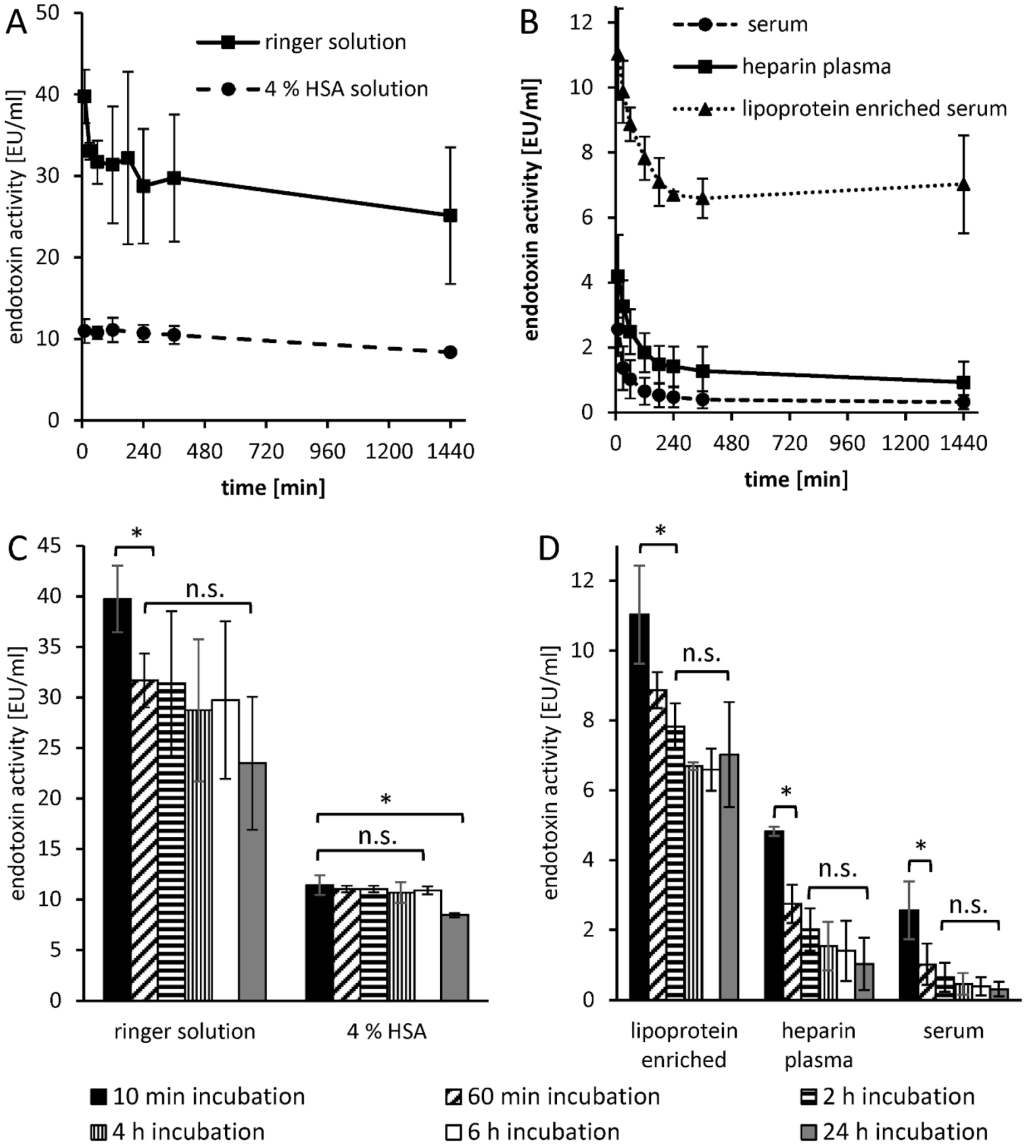
Endotoxin neutralization as a function of incubation time in different media. To check whether endotoxin neutralization in different media is time-dependent, 5 ng/mL LPS (*E. coli*) were incubated in different solutions for 24 h (reverse mode) and the endotoxin recovery was measured by LAL test. The kinetic LPS neutralization was tested in ringer solution and ringer solution containing 4% (w/v) human serum albumin (**A**). Fresh human serum, heparin plasma and lipoprotein-enriched serum were also included in this study (**B**). The bars marked with * indicate from which incubation time of LPS a significant (p ≤ 0.05) endotoxin reduction is achieved compared to the 10 min sample (C and D). n.s. not significant. Mean ± standard deviation of three independent experiments are shown.

For time dependent endotoxin recovery studies, 5 ng/mL LPS (*E. coli*) was incubated in undiluted samples for different time periods (reverse mode). A significant (p ≤ 0.05) reduction of the endotoxin activity between 10 min and 24 h incubation could be measured in all tested media (Fig. 8C,D). In protein-free ringer solution, the greatest decrease in endotoxin activity was measured between the 10 and 60 min incubated samples (Fig. 8A). A significant (p ≤ 0.05) difference in LPS recovery between samples, incubated longer than 60 min, was not observed (Fig. 8C). In HSA solution, only the 24-h incubated endotoxin showed a significantly (p ≤ 0.05) lower LPS recovery (Fig. 8A,C). In heparin plasma, endotoxin recovery was 296 ± 61% higher over the entire incubation period compared to serum. In lipoprotein-enriched serum the endotoxin recovery was even 1305 ± 626% higher than in serum. The strongest endotoxin neutralization in lipoprotein-enriched serum takes place in the first 2 h of incubation (Fig. 8B). A significant (p ≤ 0.05) reduction in LAL levels between 2 h incubation and 24 h incubation was not observed (Fig. 8D). The measured kinetic decrease of endotoxin recovery was similar in heparin plasma and serum. The strongest LPS neutralization occurred in the first 2 h. During the remaining incubation period, a further decrease was observed, but this was not significant (p ≤ 0.05) with the available measurement data.

### Influence of citrate anticoagulation on endotoxin activity in human plasma

**Figure 9:**
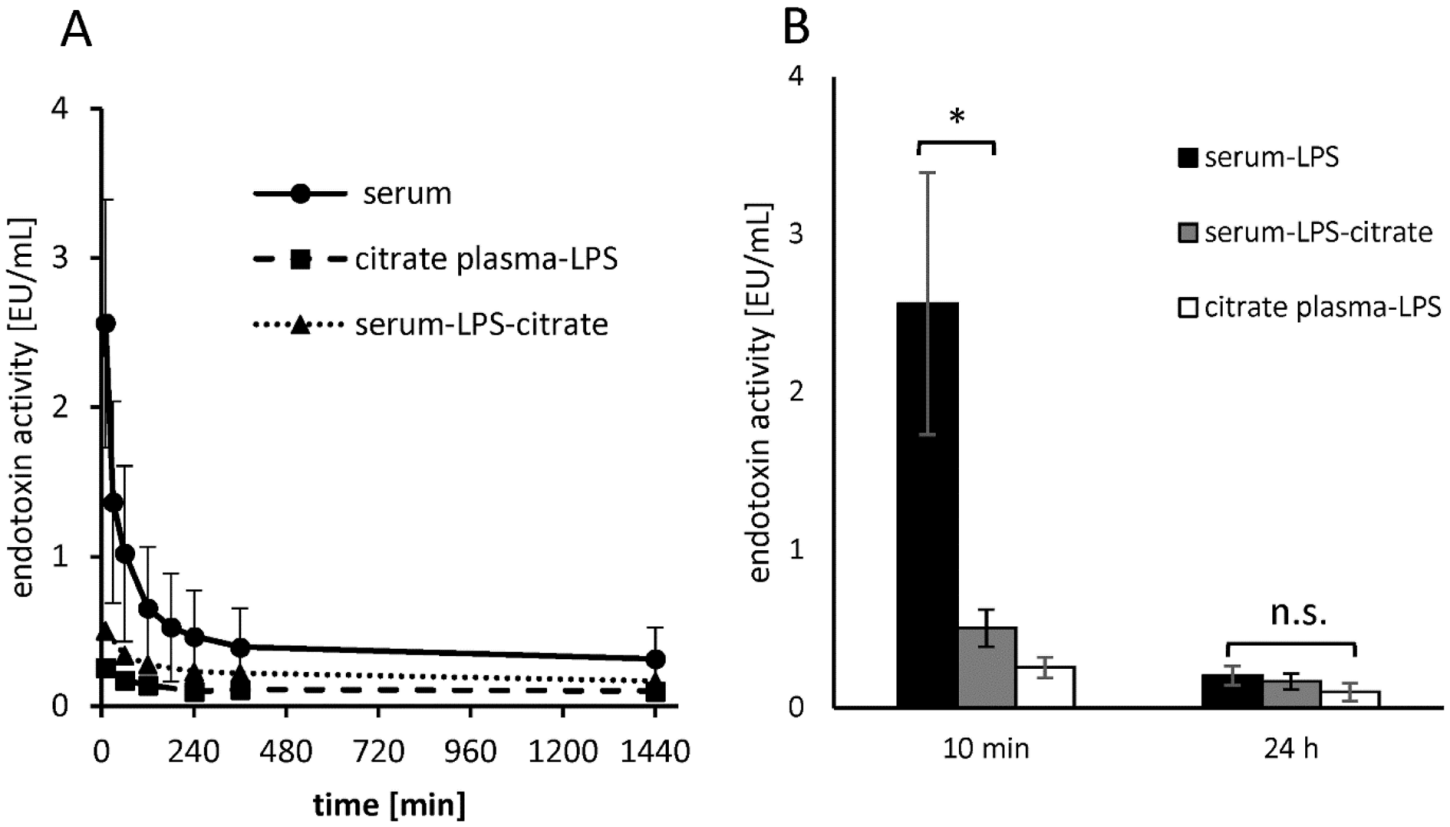
Influence of citrate anticoagulation on endotoxin activity in human plasma. To check whether the time-dependent endotoxin neutralization of 5 ng/mL LPS (*E.coli*) is influenced by citrate anticoagulation, LPS was incubated in different plasma and serum samples for 24 h (reverse mode) and the endotoxin recovery was measured by LAL test (**A**). The kinetic LPS neutralization was tested in citrate plasma (citrate plasma-LPS), in serum and in serum where citrate was added after the incubation period immediately before LAL analysis (serum-LPS-citrate). The bars marked with * indicate from which incubation time of LPS a significant (p ≤ 0.05) endotoxin reduction is achieved compared to the 10 min sample (**B**). n.s. not significant. Mean ± standard deviation of three independent experiments with plasma/serum from different donors are shown.

To determine the extent to which citrate anticoagulation affects endotoxin neutralization in human plasma, a time-dependent recovery study of endotoxins in citrated plasma was performed. The time-dependent endotoxin neutralization was compared between citrated plasma (citrate plasma-LPS), serum and serum where citrate was added after the incubation period (serum-LPS-citrate) immediately before LAL analysis. Since LAL analysis requires a free calcium level of 1 mM for maximum endotoxin recovery, the citrate-containing plasma and serum samples were diluted 1:10 with ringer solution (2.25 M iCa) instead of water before LAL measurement was performed. The iCa levels of the diluted samples were measured with an ionometer and were 0.97 ± 0.07 mM for citrate plasma and 1.13 ± 0.02 mM for citrated serum. The results show that the endotoxin recovery of 5 ng/mL LPS after a 10 min incubation period in citrated plasma is 89 ± 5.5% lower than in serum. When citrate is added in serum after the LPS incubation, the endotoxin recovery is 78.7 ± 8.8% lower than in serum. Prolonged incubation of LPS in plasma and serum samples decreases endotoxin recovery in all samples (Fig. 9A). The strongest decrease in endotoxin recovery is observed in serum. In the citrate containing samples, a very low endotoxin recovery is already present from the beginning, whereas it makes a difference whether citrate is present before or after the LPS addition. If LPS is added to citrated plasma, the endotoxin recovery is lower than if citrate is added to the LPS containing serum. After a 24-h incubation period of LPS in serum or citrated plasma, the endotoxin recovery is similarly low in all samples. A significant (p ≤ 0.05) difference in endotoxin activity after 24 h incubation time was not measured between serum and citrated plasma (Fig. 9B). This means that the time-dependent endotoxin neutralization effect in human plasma is accelerated by citrate anticoagulation.

**Figure 10:**
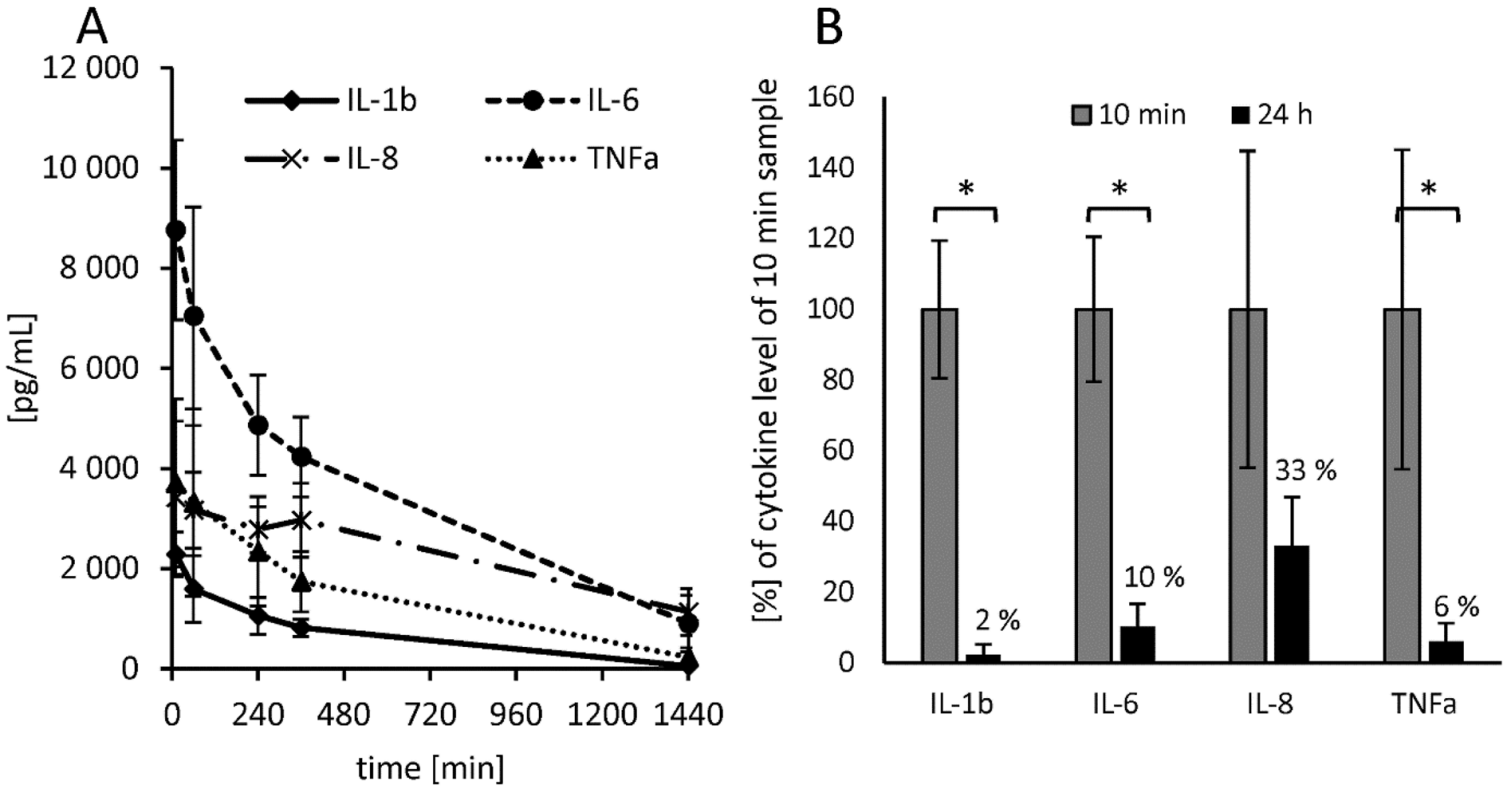
Effect of endotoxin neutralization on monoocyte activation in whole blood. Therefore, 2 ng/mL LPS (*E. coli*) were incubated in fresh heparin plasma for different time periods (reverse mode). All plasma samples were then mixed with blood cells from the same donor in a 1:1 ratio and incubated at 37 °C for 4 h. After incubation cytokine levels were determined by ELISA. Mean values ± standard deviation are shown (**A**). The bars marked with * indicate a significant (p ≤ 0.05) reduction of secreted cytokine levels in blood between 10 min and 24 h LPS pre-incubation (**B**).

To check whether the time-dependent neutralization of endotoxins in human plasma also influences the stimulating properties on monocytes, separate tests were performed. LPS molecules masked by plasma components show not only lower LAL readings but also much lower activation properties of monocytes (Fig. 10A). A 24-h pre-incubation of LPS in human plasma lead to a significant (p ≤ 0.05) reduction of cytokine secretion by monocytes compared to the 10-min pre-incubation. For IL-1β, endotoxin neutralization resulted in a reduction of 98 ± 3%, TNF-α levels were reduced by 94 ± 5% and IL-6 levels by 90 ± 6%. Only the IL-8 values did not decrease significantly (p ≤ 0.05) by pre-incubation of LPS in plasma (Fig. 10B).

### Influence of lipoproteins on endotoxin recovery

A possible explanation for the neutralization of endotoxins in human blood is described by the endotoxin-lipoprotein hypothesis, established by Rauchhaus et al.^19^. In order to find out to what extent lipoproteins are responsible for endotoxin neutralization in human blood, lipoprotein-enriched and lipoprotein-depleted serum was prepared. Lipoprotein-enriched serum was prepared by hemodiafiltration using the Albuflow filter. Lipoprotein and protein content of native, lipoprotein-enriched, and lipoprotein-depleted serum were measured with the Cobas c311 analyzer device with according test reagents and are given in Table 2.

**Table 2:** Measured lipoprotein (LDL, HDL), albumin and total protein concentrations from native, lipoprotein-enriched and lipoprotein-depleted serum.

	LDL (mg/dL)	HDL (mg/dL)	Albumin (g/dL)	Total protein (g/dL)
Serum native	96	36.3	3.8	5.59
Lipoprotein-depleted serum	9	22.8	2.79	3.85
Lipoprotein-enriched serum	881	146.2	2.88	8.26

**Figure 11:**
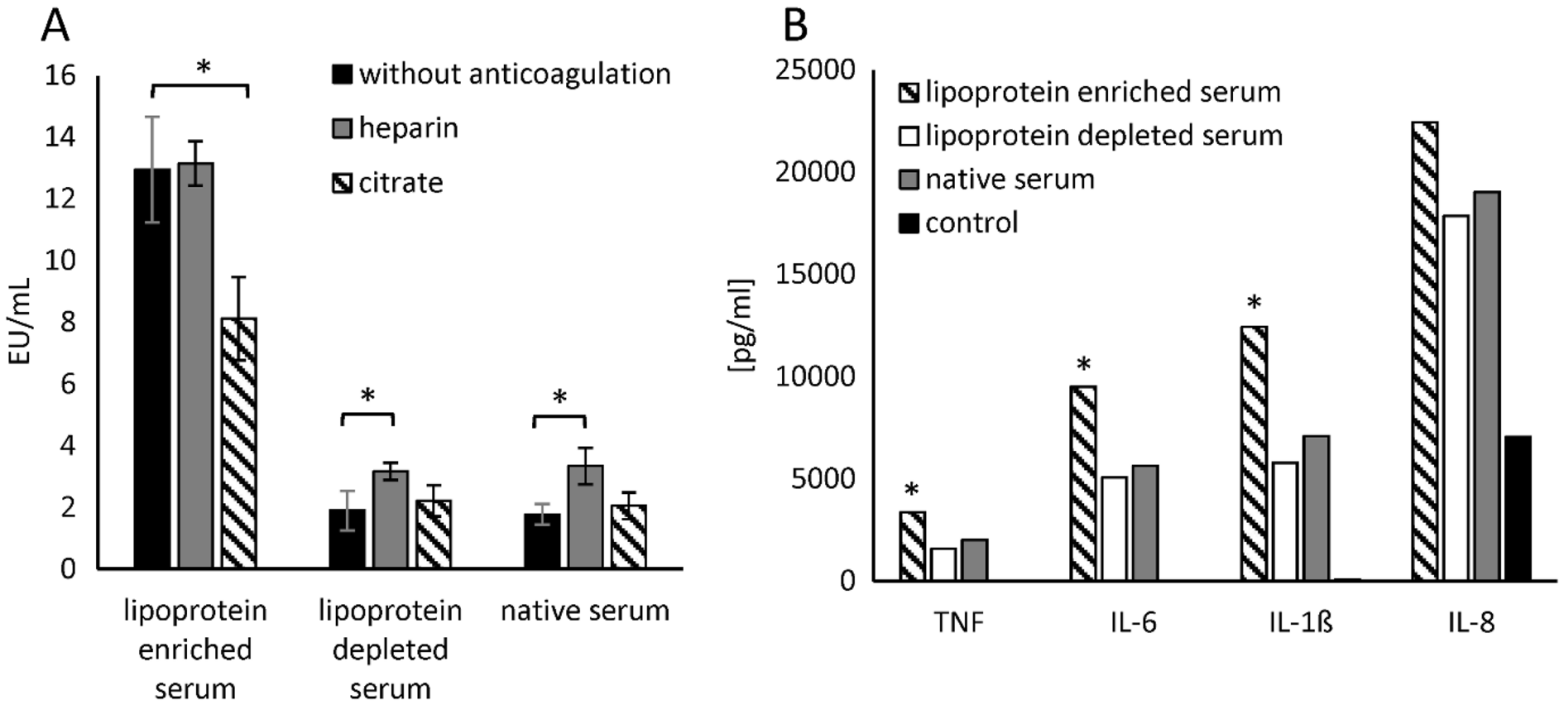
(**A**) Endotoxin recovery between native serum, lipoprotein-depleted and lipoprotein-enriched serum in dependence of anticoagulation. Native serum, lipoprotein-enriched and -depleted serum was spiked with heparin (10 IU/mL), citrate (9 mM) or ringer solution for volume compensation in samples without anticoagulation. Endotoxin recovery of 5 ng/mL LPS (*E. coli*) was determined in the different serum samples. Mean ± standard deviation of three independent experiments are shown. The bars marked with * show a significantly (p < 0.05) difference in endotoxin activity compared to serum without anticoagulants. (**B**) Monocyte stimulation experiment with LPS, which was incubated in different plasma fractions. LPS was incubated in native serum, lipoprotein-enriched serum and lipoprotein-depleted serum for 24 h. Freshly collected serum blood was spiked (1 ng/mL) with these differently pre-incubated endotoxins. After 4 h incubation at 37 °C the serum levels of TNF-α, IL-1β, IL-6 and IL-8 were determined to test the biological activity of the different pre-incubated endotoxins.

The next step was to determine the LPS recovery of 5 ng/mL LPS (*E. coli*) between native, lipoprotein-enriched and lipoprotein-depleted serum as a function of anticoagulation. Surprisingly, an increased LPS recovery was observed in lipoprotein-enriched serum. The addition of heparin did not improve the recovery, whereas citrate significantly (p ≤ 0.05) reduced the LAL value by 37 ± 10% (Fig. 11A). LPS recovery without anticoagulation was 86 ± 5% lower in native and lipoprotein-depleted serum than in lipoprotein-enriched serum. In native and lipoprotein-depleted serum, heparin significantly (p ≤ 0.05) increased endotoxin recovery between 67 ± 19 and 88 ± 14%, while citrate did not significantly (p ≤ 0.05) alter the LAL measurement. In order to check whether the biological activity of endotoxins is influenced by lipoproteins in addition to the LAL activity, a monocyte stimulation experiment was performed. Serum blood was prepared from 3 donors and stimulated with 1 ng/mL LPS. The LPS used for the stimulation was incubated for 24 h in native serum, lipoprotein-enriched serum and lipoprotein-depleted serum. After 4 h incubation of the LPS-spiked blood samples, IL-6, IL-8, IL-1β and TNF-α were quantified in blood. Serum blood without endotoxin was used as negative control. The results confirmed the LAL results (Fig. 11B). LPS incubated in the high molecular weight (lipoprotein-enriched) serum fraction showed a significantly (p ≤ 0.05) higher monocyte activation (higher cytokine secretion, except IL-8) than LPS incubated in native or low molecular weight (lipoprotein-depleted) serum. This also means that lipoprotein is not responsible for the endotoxin neutralization in the LAL test and also not for the inactivation of the monocyte stimulating property of endotoxins. Rather, small to medium molecular weight plasma components appear to be responsible for endotoxin inactivation in plasma.

**Figure 12:**
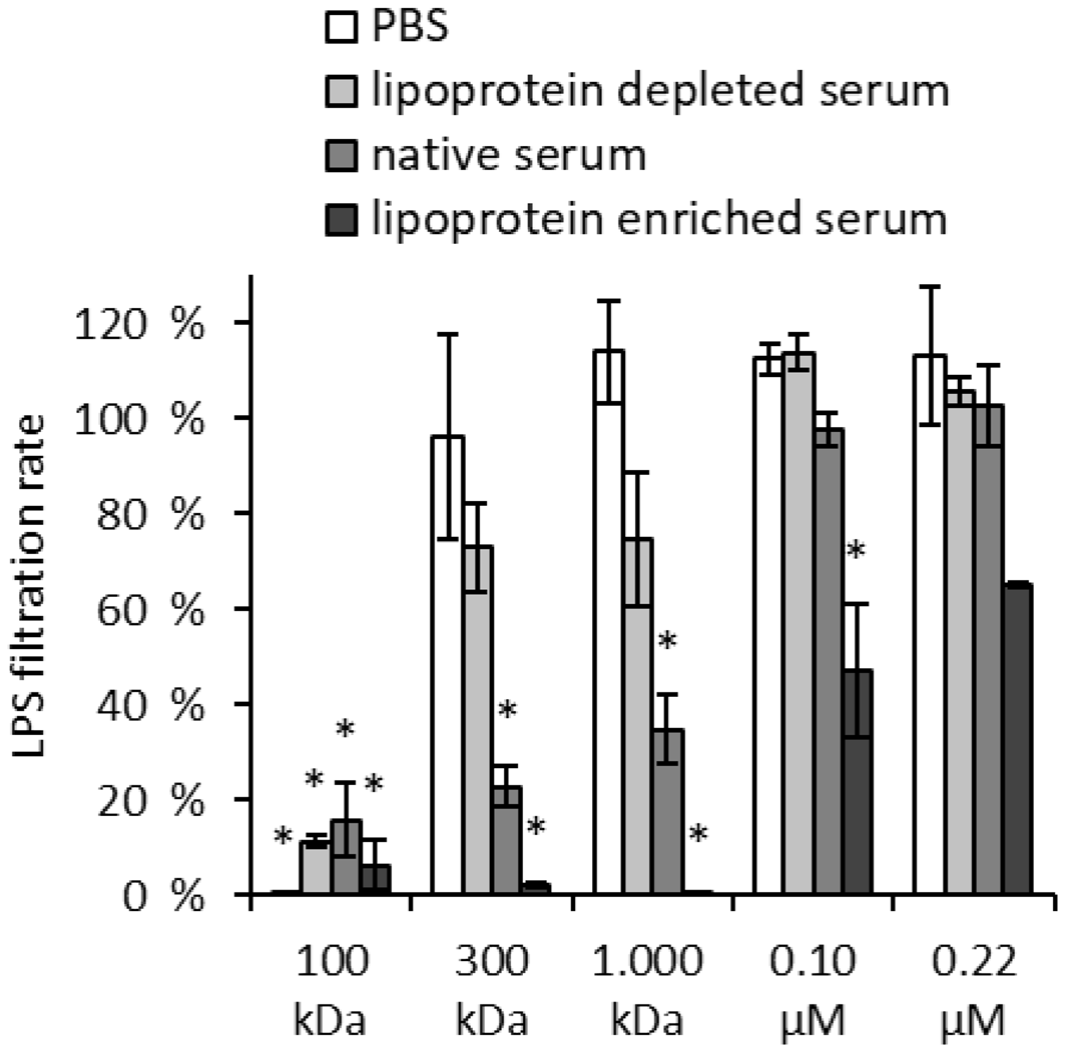
Influence of lipoproteins on the filtration rate of endotoxin. 5 ng/mL LPS (*E. coli*) were dissolved in PBS, lipoprotein-depleted serum, native serum and lipoprotein-enriched serum and filtered through spin columns with 100, 300 and 1000 kDa MWCO. Additionally, the samples were filtered through a 0.1 µm and 0.22 µm sterile filter. The figure shows the percentage of endotoxin values measured in the filtrate samples compared to the unfiltered samples (N = 3).

To check whether endotoxins have an affinity to plasma lipoproteins and bind to them, filtration tests were performed. Since lipoproteins have a diameter between 7 (high density Lipoprotein, HDL) and 200 nm (chylomicron)^46^, the filtration rate of LPS should be reduced when bound to the lipoproteins. The filtration rates of LPS dissolved in PBS, in lipoprotein-enriched serum, in native serum and in lipoprotein-depleted serum were determined by membranes with 100, 300 and 1000 kDa molecular cut off (MWCO) and by sterile filters with 0.1 and 0.22 µm pores. The endotoxin quantification was determined in the respective filtrate by the LAL test and expressed as a percentage of the endotoxin values in the unfiltered sample (Fig. 12). The results show that LPS, when dissolved in PBS or in lipoprotein-depleted serum, is retained only by the filter with 100 kDa molecular cut off. In native serum, the filtration rate of LPS through the 1,000 kDa MWCO membrane was significantly (p < 0.05) lower and in lipoprotein-enriched serum a significantly (p < 0.05) lower filtration rate of LPS could be detected even with the 0.1 µm filter compared to the respective unfiltered sample.

The results of the filtrate tests show that HDL and LDL are filtered unhindered through the membrane of the sterile filter with 0.1 µm pores, whereas no lipoproteins can be filtered through the membrane with 100 kDa MWCO (Table 3). With LPS, a 100% filtration rate could not even be achieved with the 0.2 µm sterile filters. A possible cause could be that some of the endotoxins bind to chylomicrons, which can reach a size of more than 0.2 µm. Another explanation could be that endotoxin micelles can reach a size that makes filtration through sterile filters difficult.

**Table 3:** HDL and LDL values in serum and serum filtered through membranes with different pore sizes. Mean ± standard deviation of three independent experiments with serum from different donors (N = 3) are shown. The values marked with * indicate measured values below the detection limit of the test kit.

	Unfiltered serum	Filtered serum				
		100 kDa	300 kDa	1000 kDa	0.1 µm	0.22 µm
HDL	53 ± 46	< 3 mg/dL*	13 ± 11	17 ± 14	53 ± 46	53 ± 46
LDL	142 ± 148	< 3 mg/dL*	28 ± 30	41 ± 42	143 ± 149	143 ± 148

### Filtration rate of endotoxin neutralization substances in plasma

**Figure 13:**
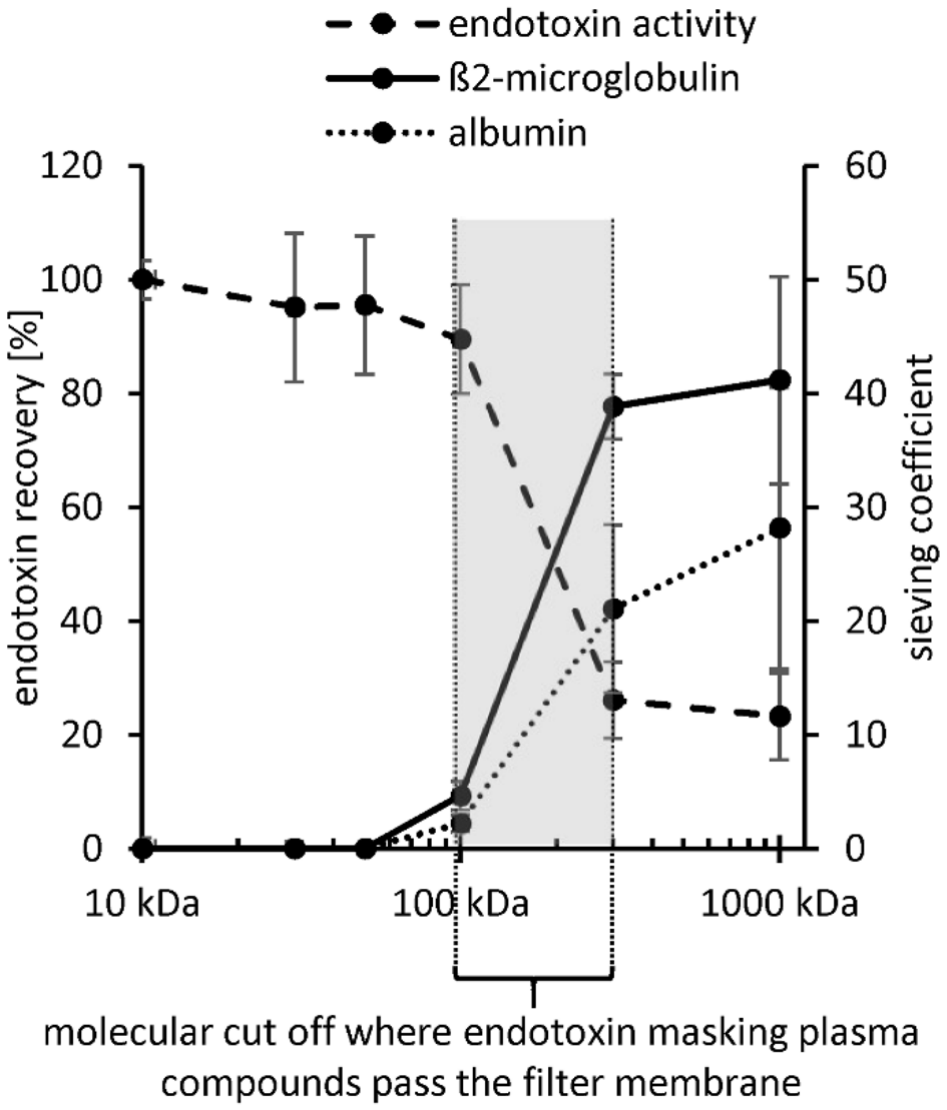
Determination of the filtration rate of endotoxin neutralizing substances in plasma. Fresh serum was spiked with 3 µg/mL β2-microglobulin (β2-MG) and subsequently filtered through spin columns with membranes of different molecular cut off. The sieving coefficient of β2-MG and albumin was then determined in the respective filtrate. To determine the filtration rate of the endotoxin neutralizing plasma components, the endotoxin recovery of 5 ng/mL LPS (*E. coli*) was measured in the filtrate samples. Membranes with the following molecular cut offs were used: 10, 30, 50, 100, 300 and 1000 kDa. Mean ± standard deviation of three independent experiments with serum from three different donors are shown.

Filtration tests were performed to determine the approximate molecular size of the endotoxin-neutralization plasma components. Fresh serum from different donors was filtered through spin columns with membranes of different molecular cut off. Endotoxin recovery tests were performed with the different filtrates. In order to have a size comparison, the sieving coefficient of albumin (66 kDa) and β2-microglobulin (β2-MG, 11.7 kDa) was determined in the filtrate samples. The results show that endotoxin neutralization was measurable in 100 kDa filtrate. In the 300 kDa filtrate the endotoxin recovery was only 26 ± 7%, indicating that the endotoxin neutralization plasma substances are filtered through this membrane. The sieving coefficient of β2-MG behaves similarly (Fig. 13). This indicates that endotoxin neutralization substances have a molecular weight similar to β2-MG, namely 12 kDa.

### Influence of monocytes on endotoxin activity in blood

Endotoxins are known to activate complement, the kinin system, monocytes, platelets and endothelial cells and activate the innate immune system. In blood, monocytes react very sensitively to the presence of endotoxins. These carry the toll like receptor 4 which is a transmembrane protein. Its activation leads via intracellular signaling pathway NF-kB to inflammatory cytokine production. In our study, we investigated if leucocytes influence the endotoxin serum activity. Therefore, we incubated 5 ng/mL LPS (*E. coli*) in heparinized whole blood and in blood in which the leucocyte count (buffy coat) was strongly reduced. To eliminate the influence of heparin, the same experiment was performed with serum blood and leucocyte-depleted serum blood (Table 4).

**Table 4:** Blood cell count of blood and leucocyte-depleted blood and serum blood. Leucocyte-depleted whole blood was prepared by removing the buffy coat after centrifugation. Serum blood was prepared by mixing serum and washed blood cells from the same donor. The number of white blood cells (WBC), red blood cells (RBC) and hematocrit (HCT) were determined using a blood cell counter.

	Serum blood		Whole blood	
	− Buffy coat	+ Buffy coat	− Buffy coat	+ Buffy coat
WBC × 10^3^/µL	0.5 ± 0.2	5.6 ± 0.2	0.5 ± 0.2	5.2 ± 0.5
RBC × 10^6^/µL	5.2 ± 0.1	5.1 ± 0.2	5.2 ± 0.2	5.1 ± 0.3
HCT (%)	45.4 ± 1.4	44.1 ± 1.8	44.5 ± 0.9	43.7 ± 1.7

**Figure 14:**
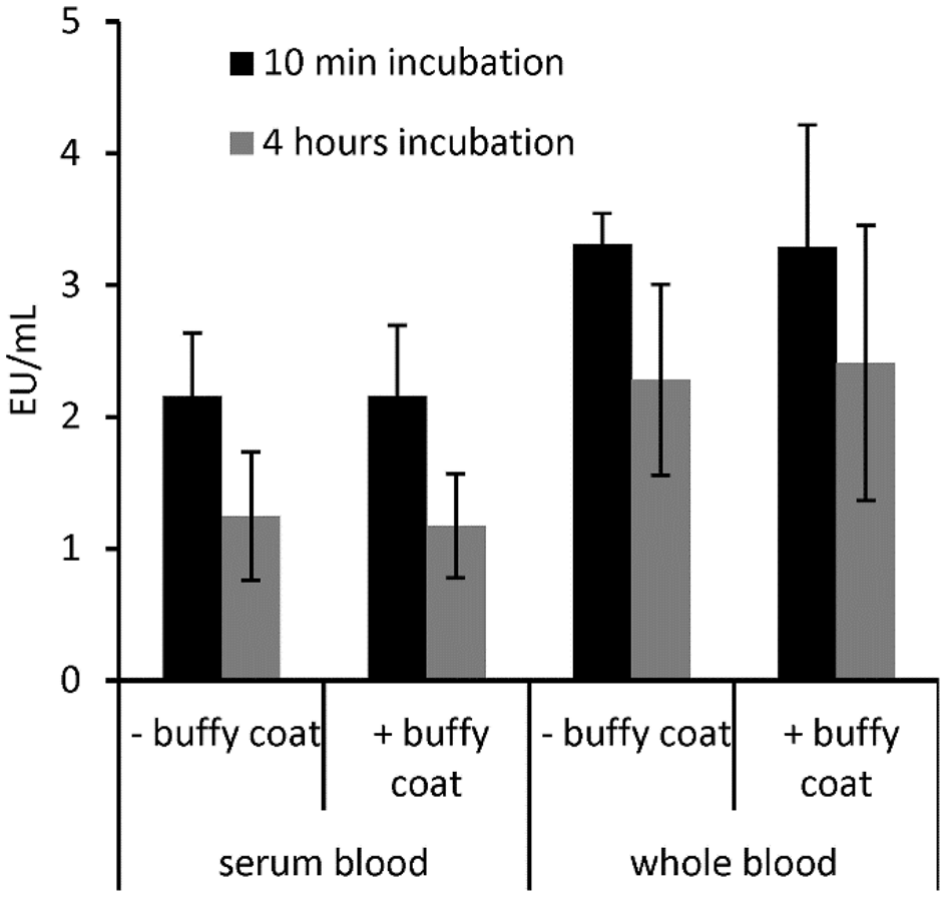
Influence of leucocytes on endotoxin activity in blood. It was examined whether the incubation of LPS with leucocytes has an influence on the endotoxin activity. This was performed with heparinized whole blood as well as with serum blood without anticoagulant. Blood with normal leucocyte count is labeled with “+ buffy coat”, leucocyte-depleted blood is labeled as “− buffy coat” in the graph. Mean ± standard deviation of three independent experiments with blood from different donors are shown.

The results show that leucocyte-depletion does not affect the endotoxin content in serum or plasma. Even after a 4-h incubation with the blood cells no significant (p ≤ 0.05) difference could be measured (Fig. 14). This leads to the conclusion that plasmatic and non-cellular components in the blood are responsible for endotoxin neutralization.

### Endotoxin neutralization by heparin binding plasma compounds

**Figure 15:**
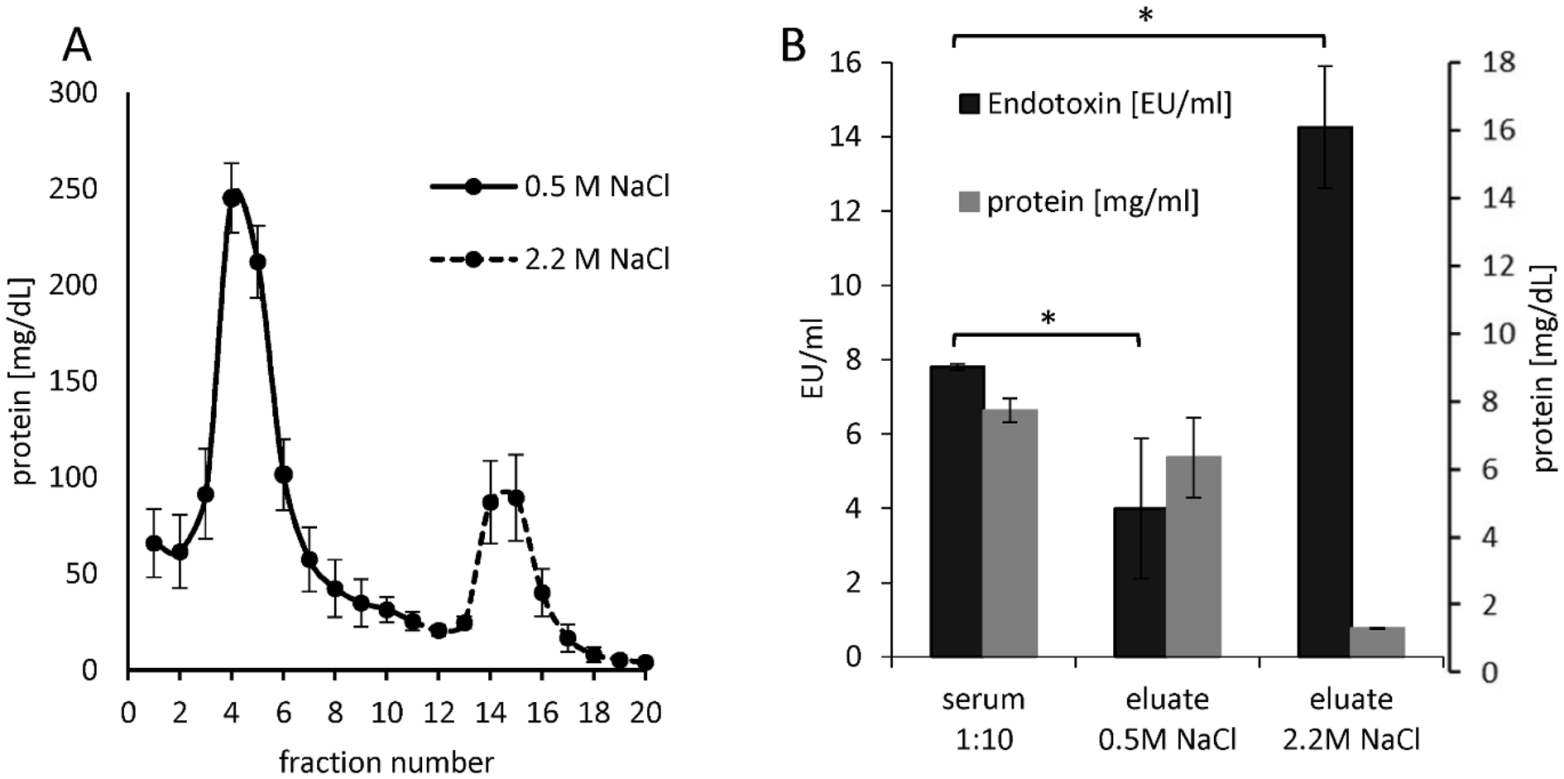
Separation of heparin-binding plasma substances and testing for their ability to mask endotoxins. 25 mL freshly donated serum was pumped through a 5 mL column filled with heparin-immobilized adsorbent at a flow rate of 0.5 mL/min. After washing the column with physiological saline solution, heparin-binding plasma components with medium affinity were eluted using 10 mL 0.5 M NaCl solution. The substances strongly bound to heparin were eluted with 2.2 M NaCl solution. The eluates were separated into 1 mL fractions and their protein content was determined (**A**). The respective fractions of the two eluates were pooled and transferred into ringer solution. An endotoxin recovery of 5 ng/mL LPS (*E. coli*) in the two eluates and in serum diluted 1:10 with ringer solution was then performed using the LAL test (**B**). The bars marked with * indicate a significant (p < 0.05) different in endotoxin recovery. Mean ± standard deviation of three independent experiments with serum from three different donors are shown.

In the literature, it is pointed out that, the LPS activity in plasma also depends on the anticoagulant. Especially blood anticoagulated with citrate or EDTA shows a reduced endotoxin recovery^39,40^. To the best of our knowledge our study was the first to show that heparin significantly (p ≤ 0.05) improves endotoxin recovery in human plasma. Furthermore, it could be shown that plasma components with small to medium molecular weight are responsible for endotoxin neutralization. In order to confirm that some of the endotoxin neutralization substances in plasma have an affinity to heparin, an attempt was made to enrich heparin-binding substances from freshly obtained human serum using a column filled with heparin-immobilized adsorbent. Heparin-bound plasma components were eluated with 0.5 M and 2.2 M NaCl solutions to separate the adsorbent bound plasma substances into medium and high heparin affinity compounds. The eluates were separated into 1 mL fractions and the respective protein content was measured. The largest amount of heparin-bound plasma proteins could already be eluted with 0.5 M NaCl solution. The amount of proteins strongly bound to heparin, which were eluted with 2.2 M NaCl, was much lower (Fig. 15A). The two heparin-binding protein fractions (0.5 M and 2.2 M NaCl) were then transferred to ringer solution using spin concentrators. Serum diluted 1:10 with ringer solution and the two heparin-binding fractions were then mixed with 5 ng/mL LPS (*E. coli*) and the endotoxin recovery was determined by LAL test. The 1:10 dilution of serum was performed to obtain similar protein contents as in the two heparin-binding protein fractions. The results show that heparin-binding plasma components eluted with 0.5 M NaCl result in significantly (p < 0.05) higher endotoxin neutralization than native serum (Fig. 15B). These results show that heparin-immobilized sepharose binds endotoxin-neutralization substances from serum, which can be eluted using 0.5 M NaCl solution. This could also explain why the endotoxin recovery in heparin plasma is significantly higher than in serum. Heparin in plasma binds endotoxin neutralization substances and thus increases endotoxin recovery.

**Figure 16:**
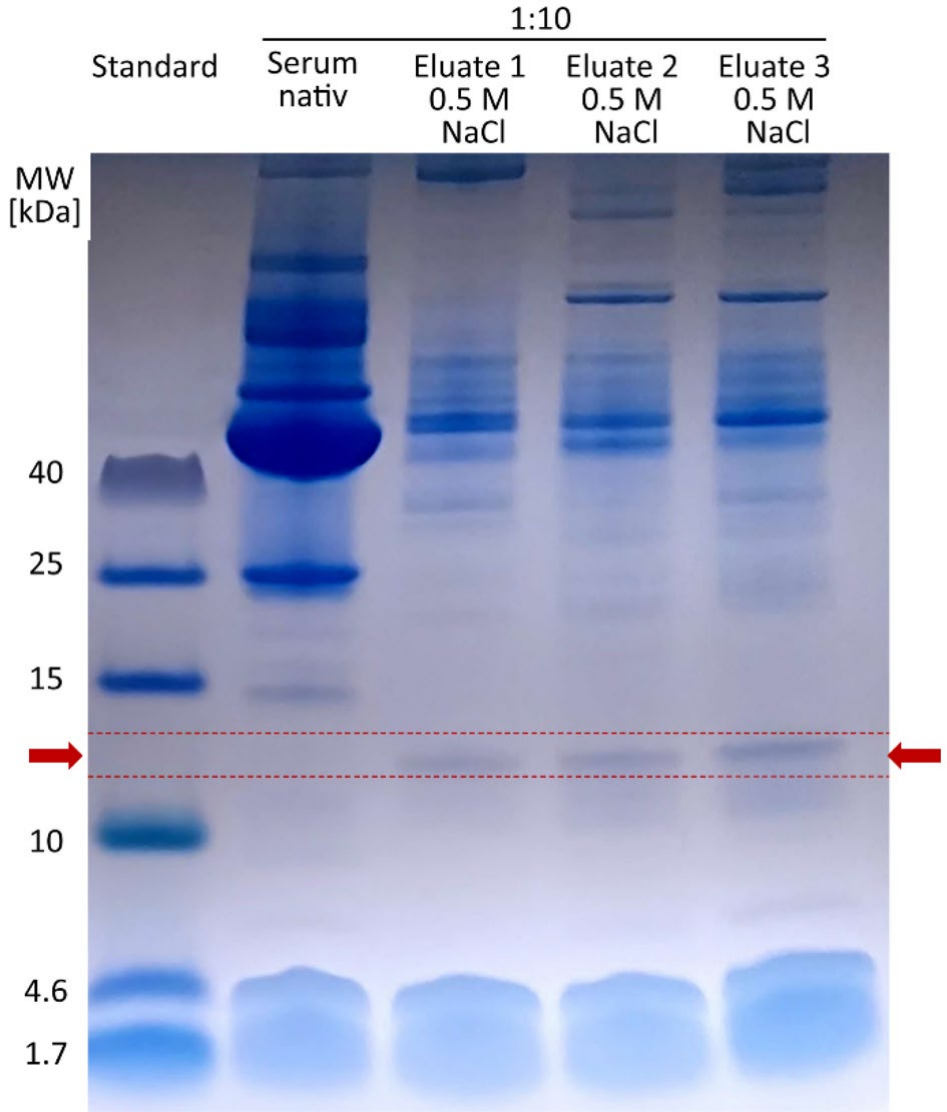
Protein separation of heparin-binding proteins by SDS page. The heparin-binding proteins, which were eluted with 0.5 M NaCl and showed strong endotoxin neutralization, were applied to the gel together with diluted serum. A protein band with a molecular mass between 12 and 13 kDa is only visible in the eluates and marked with red arrows in the graph.

The proteins of the endotoxin neutralization eluate were separated according to their mass using sodium dodecyl sulfate polyacrylamide gel electrophoresis (SDS-PAGE) and detected by coomassie blue staining (Fig. 16). The heparin binding protein fraction eluted with 0.5 M NaCl shows a protein band with an approximate molecular mass between 12 and 13 kDa, which is not detectable in native serum sample. This small protein was concentrated using the heparin immobilized adsorbent column and could be responsible for endotoxin neutralization.

## Discussion

The Limulus Amebocyte Lysate (LAL) test is a common method for the detection of LPS. Both the endotoxin and the LAL reagent are subject to biological fluctuations. A significant variation in the reactivity of the LAL cascade on different LPS sources was observed. Studies in human volunteers have shown that the pyrogenic threshold doses for endotoxin from *Salmonella typhi, Escherichia coli* and *Pseudomonas aeruginosa* are approximately 0.1, 1.0 and 60 ng/kg, respectively. Due to the strong neutralization of circulating endotoxins in human blood, reproducible quantification of endotoxins by LAL assay is currently not possible. In human plasma, endotoxin neutralization is partly caused by antimicrobial peptides and proteins (AMPs), or by other cationic molecules that bind to the anionic domain of LPS and reduce or eliminate their biologic activity. The binding of AMPs to lipopolysaccharide is a crucial step of the innate immune system both for their antimicrobial effect and for their immunomodulatory properties. On the one hand, killing Gram-negative bacteria by AMPs can be an effective strategy to prevent the spread of bacteria in the human body, which can lead to septic shock. On the other hand, by neutralizing circulating endotoxins, AMPs can successfully reduce the production of nitric oxide and pro-inflammatory mediators, thus preventing severe tissue damage^47^.

**Figure 17:**
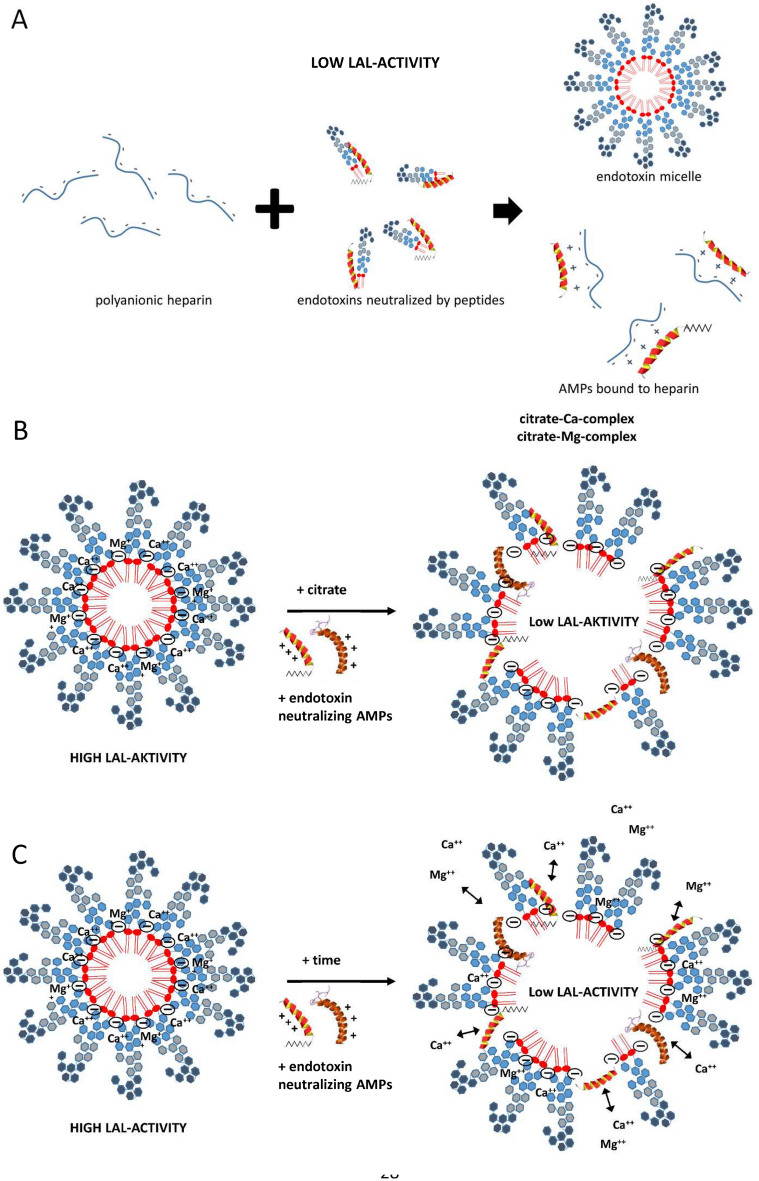
Schematic representation of endotoxin neutralization by plasmatic AMPs and its influence by anticoagulation. The polyanionic heparin molecule binds the cationic AMP molecules by ionic interaction. The formation of a heparin-AMP complex overrides the endotoxin neutralization effect of some AMPs (**A**). The anticoagulant citrate binds divalent cations, which are responsible for the stabilization of the LPS micelles. This leads to a better and faster penetration of endotoxin binding and neutralizing substances to the active lipid A portion of the LPS monomer resulting in lower endotoxin recovery in the LAL test (**B**). Cationic antimicrobial peptides, can diffuse into the interior of the LPS micelles and displace the divalent cations there. This leads to a destabilization of the endotoxin micelles and to a neutralization of the endotoxin activity (**C**).

In this study, we could show that the recovery of endotoxins in the blood by LAL test is influenced by various factors. Compared to aqueous solutions, which do not contain any substances interfering with the LAL test, the recovery of 5 ng/mL LPS (*E. coli*) in human serum is only 13 ± 4%. In citrate anticoagulated plasma an even lower endotoxin recovery was measured. Citrate binds divalent cations, which are responsible for the stabilization of the LPS micelles. This leads to a better and faster penetration of endotoxin binding and neutralization substances to the active lipid A portion of the LPS monomer. Interestingly, we could show that the endotoxin neutralization effect in human plasma can be attenuated by the anticoagulant heparin. The reason for this observation could be that the polyanionic heparin binds the strongly positively charged antimicrobial peptides and thus reduces their LPS neutralizing effect (Fig. 17A). That heparin can neutralize the antimicrobial effect of synthetic cationic LF11 based AMPs has already been proven in a previous study^38^. Both unfractionated and fractionated (low molecular weight heparin) caused increased endotoxin recovery in plasma. The polyanionic anticoagulant fondaparinux (FXa inhibitor) showed no influence on endotoxin recovery in the LAL test, which shows that the sole polyanionic charge of a molecule is not responsible for influencing endotoxin activity. The fact that heparin directly affects endotoxin activity and not the LAL analysis method has been demonstrated using a blood cell model. In our blood cell model, it was shown that from 2.5 IU/mL heparin, a significant (p ≤ 0.05) increase in pro-inflammatory cytokines was secreted by the monocytes. Heparin anticoagulation not only increases the endotoxin activity in the LAL test, but also the stimulating effect on monocytes. It can be concluded that the endotoxin neutralizing and modulating effect in human plasma is attenuated by heparin anticoagulation. In contrast to heparin, citrate anticoagulation of blood significantly reduces endotoxin recovery in plasma. Citrate complexes divalent cations, which are located between the phosphate groups of the lipid A fraction and have the task of stabilizing the LPS micelles or the outer membrane of Gram-negative bacteria. The removal of the divalent cations leads to destabilization of the LPS micelles and the outer membrane of Gram-negative bacteria. The outer membrane of Gram-negative bacteria serves as the first barrier that AMPs of our innate immune system encounter. They have to cross this barrier to reach the inner cytoplasmic membrane. Initially, the peptides interact with the outside of the LPS and displace the divalent cations. The divalent cations partially neutralize the negative charge of the LPS and stabilize the outer membrane or LPS micelles^48–50^. The process of destabilization of the outer membrane by AMPs can be accelerated by complexing divalent cations with citrate. In the case of LPS micelles, endotoxin neutralization by plasma AMPs can be achieved very quickly by adding citrate, since the diffusion barrier is broken by the removal of the divalent cations (Fig. 17B). Reich et al. published the fact that citrate or other complexing agents destabilize the endotoxin micelles and thus accelerate the endotoxin neutralization by surfactants in aqueous solutions. He also describes that this neutralizing effect cannot be reversed by the addition of divalent ions^41^. It should also be noted that high citrate concentrations can directly inhibit the LAL cascade, which requires magnesium or calcium as a cofactor. However, there is sufficient magnesium in the LAL reagent and by diluting the plasma samples prior to LAL measurement, the inhibitory effect of citrate is cancelled. Recent studies have shown that some AMPs also have the potential to neutralize LPS-induced endotoxic effects. These peptides are important components of the innate defense system of all types of life^30^. They are produced in large quantities at the site of infection and/or inflammation and act rapidly against microbes^31^. Some studies also showed a direct correlation between the ability of AMPs to bind LPS and their antimicrobial activity^51–53^. One of the best-known examples of natural human AMPs that have shown high affinity to LPS in in vitro and animal models are CAP37, LL-37 and LF11 peptides. In separate experiments it was investigated which level of divalent cations is necessary for maximum endotoxin recovery. In physiological saline solution, where no substances are present that bind divalent cations and influence the ion balance, no additional dosage of calcium or magnesium ions is necessary, because the LAL reagent contains enough divalent cations for an optimal reaction of the LAL cascade. When detecting endotoxins in albumin containing solutions, it should be noted that albumin molecules bind calcium ions. At a plasma pH of 7.4, each gram albumin binds 0.8 mg calcium. This bond is dependent on the carboxyl groups of albumin and is highly dependent on pH^54^. Due to the rather low calcium binding of HSA, already 0.5 mM iCa in the albumin solution is sufficient to achieve maximum endotoxin recovery in the LAL test. For magnesium, 1 mM is necessary. In serum, 1 mM iCa or more than 2 mM iMg are required for maximum endotoxin recovery. In plasma and serum samples, the addition of divalent ions prior to LAL measurement is not necessary because the physiological iCa value in human blood is 1.2 mM and for iMg 0.5 mM. Citrate as anticoagulant should be avoided for LAL measurements, as it binds divalent ions by complex formation. A possible explanation for endotoxin neutralization in human plasma is described by the endotoxin-lipoprotein hypothesis. To test this hypothesis, a special plasma filter (Albuflow filter) was used to prepare lipoprotein-depleted and lipoprotein-enriched serum. The membranes of the Albuflow filter have a molecular cut off of 250 kDa. The produced plasma filtrate therefore contains all small to medium molecular weight plasma components. The lipoprotein-enriched serum includes all high molecular weight plasma component. When determining the endotoxin recovery by LAL test, we could show that the endotoxin neutralization in lipoprotein-enriched serum is significantly (p ≤ 0.05) lower than in native serum or in lipoprotein-depleted serum. A difference in endotoxin recovery by LAL test between native and lipoprotein-depleted serum could not be measured. This indicates that lipoproteins are hardly involved in endotoxin neutralization in our experiment setup. Possibly, the low to medium molecular weight plasma fraction is necessary for the neutralization of endotoxins by lipoproteins. It could be that the LPS aggregates are first destabilized by antimicrobial plasma components and only then, the attachment of LPS molecules to lipoprotein can take place. Barcia and Harris have hypothesized that triglyceride rich lipoproteins are part of the innate immune response^55^. With this hypothesis, they explain why lipemic blood is often observed in sepsis patients. According to this hypothesis, the liver reacts after a Gram-negative infection with the production of triglyceride rich lipoproteins and the acute phase protein lipopolysaccharide binding protein (LBP). The LBP together with the soluble CD14 receptor (sCD14) is responsible for the transfer of LPS monomers to the lipoprotein surface. Finally, LPS-loaded lipoproteins are taken up by hepatocyte and LPS is removed from the bloodstream. This hypothesis might explain why we did not see endotoxin neutralization in our experiments in the LAL test as well as reduced monocyte activation in our lipoprotein -enriched plasma fraction. Cerami et al. describe another reason why lipemic plasma is observed after infection^56,57^. His studies showed that TNF-alpha secreted by macrophages after LPS stimulation blocks the production of lipoprotien lipase and other enzymes necessary for fatty acid synthesis, which ultimately leads to the accumulation of lipoproteins during bacteremia. Our lipoprotein-enriched plasma fraction consists of the high molecular weight plasma fraction, since the treatment with the Albuflow filter removed all small to medium molecular weight plasma components. This means that neither LBP (60 kDa) nor sCD14 (50 kDa) were present in the lipoprotein-enriched plasma and therefore no transfer of LPS to the lipoprotein surface was possible.

It seems that plasma components that can pass the Albuflow membrane are responsible for a direct neutralization effect of endotoxins. This is also confirmed by the endotoxin recovery study in the filtrates obtained from membranes with different molecular cut off. In these experiments it was shown that the endotoxin neutralizing plasma components are filtered through membranes with a molecular cut off between 100 and 300 kDa. Heparin increased endotoxin recovery in native and lipoprotein-depleted serum, whereas in lipoprotein-enriched serum heparin did not alter endotoxin neutralization. The filtration tests of LPS through membranes with different molecular cut off showed a significant (p ≤ 0.05) decrease of LPS filtration with increasing lipoprotein content. Even sterile filters with 0.22 µm pores showed a reduced LPS filtration even though the filtration rate of LDL and HDL was 100%. These observations suggest that LPS partially bind to chylomicrons, which can be as large as 200 nm and are therefore not completely filtered through the sterile filters. LPS incubated with chylomicrons prior to intravenous application significantly reduced mortality in a rat model compared to LPS application without pre-incubation^27^. Read published in 1993 that chylomicrons inactivate endotoxins and that after application in mice 20% of injected endotoxins were detected in bile^58^. In addition, a faster clearance of endotoxins by the liver could be observed by chylomicron administration^27^. Barcia and Harris could show that triglyceride-rich lipoproteins are part of the innate immune system^55^. They could also show that the binding of LPS to chylomicrons doubles the clearance through the liver and that the endotoxins are disposed of by hepatocytes and not by Kupffer cells. They point out that in animal models it has been shown that in LPS-induced inflammation the liver produces more triglyceride-rich lipoproteins, which could be an immune response (LPS detoxification). This effect is also known as the immunomodulatory response of the liver to infections^28^. All studies on the affinity between endotoxins and lipoproteins suggest that the interaction is based on the positively charged apolipoprotein B and the hydrophobic phospholipid layer of the lipoprotein vesicle. The lipoproteins could act as transport vesicles of endotoxins in the blood to the liver. Via the enterohepatic circulation, the endotoxin, which enters the bloodstream during leaky-gut-syndrome, is disposed of with the help of the lipoproteins in the gallbladder to the liver.

Separate experiments in our study showed that the neutralization or inhibition of endotoxins in human plasma is time-dependent. In aqueous solution, a significant (p < 0.05) change in endotoxin activity could only be detected within the first hour. In the 4% HSA solution, a significant (p < 0.05) difference in endotoxin recovery was measured only after a 24-h incubation. When LPS was incubated in serum, heparinized plasma or lipoprotein-depleted serum, a steady decrease in endotoxin recovery was observed in the first 4 h. By means of a stimulation experiment in whole blood it could be shown that not only the LAL activity but also the immunological activity of the endotoxins is indirectly proportional to the pre-incubation time in the plasma. After a 24-h pre-incubation of LPS in plasma, its immunostimulating properties on monocytes drop drastically. Through endotoxin neutralization by plasma compounds, the release of pro-inflammatory cytokines (TNF-α, IL-6 and IL-1β) of monocytes could be reduced by ≥ 90%. It can therefore be assumed that human plasma has a modulating effect on endotoxin activity to cellular immune response. LPS is a classical pathogen-associated molecular pattern (PAMP), which is immediately recognized by our innate immune system. In blood, endotoxins cause immediate activation of monocytes and macrophages, which in turn can trigger a pro-inflammatory reaction. Just as important as the sensitive and rapid recognition of potential infectious microbes is the inactivation or inhibition of such PAMPs to prevent an overreaction of our immune system (cytokine storm). According to our results, the modulation of immunologically active endotoxin is partially controlled by endotoxin-binding substances contained in human plasma (Fig. 17C). Kinetic investigations of endotoxin activity in citrate anticoagulated plasma show that endotoxin neutralization occurs significantly faster. Even the restoration of a physiological calcium level prior to LAL measurement cannot improve endotoxin recovery in citrate plasma. Thus, it could be shown that the endotoxin neutralization effect in human plasma is not reversible. After a 24-h incubation, the LAL activity in serum (no anticoagulant) was as low as in citrated plasma. It appears that the binding of the divalent cations destabilizes the endotoxin micelles and the endotoxin-binding substances can rapidly penetrate into the interior of the micelles and act there. This means that the lowering or binding of divalent cations in the blood accelerates endotoxin neutralization. In the course of this study, the extent to which leucocytes influence the LAL recovery of endotoxins in the blood was also investigated. After incubation with whole blood and leucocyte-depleted blood, no difference in endotoxin recovery could be measured in serum or heparin plasma. This indicates that LPS micelles can neither adsorb or bind to the surface of monocytes nor can they be phagocytosed by them. To determine whether heparin-binding plasma substances are actually involved in endotoxin neutralization, heparin-binding substances were separated from human plasma by affinity chromatography (heparin-immobilized sepharose). Serum components with low to medium affinity for heparin showed significantly (p > 0.05) stronger endotoxin neutralization than native serum in the determination of endotoxin recovery. This indicates that some endotoxin neutralizing plasma components have an affinity for heparin. This observation also explains the increased endotoxin recovery in human heparin anticoagulated blood. The gel electrophoresis separation of the heparin binding plasma substances showed a clear concentration of a plasma protein with a molecular weight between 12 and 13 kDa. In the endotoxin recovery study of filtrates obtained from membranes with different molecular cut offs, it was shown that the sieving coefficient of β2-microglobulin with a molecular weight of 11.7 kDa is similar to the sieving coefficient of endotoxin neutralizing plasma components. These results indicate that the concentrated protein in the eluate with a molecular weight between 12 and 13 kDa is involved in endotoxin neutralization. Whether this is a plasma protein with endotoxin-inactivating properties remains to be clarified in further investigations. In summary, we were able to show with this study that endotoxin neutralizing substances are present in human blood. This neutralization of endotoxins caused by plasma components is strongly influenced by the type of anticoagulant. While heparin significantly worsens the endotoxin inactivation by binding cationic plasma components, the time dependent endotoxin neutralization process can be significantly accelerated by citrate anticoagulant. Lipoproteins appear to bind endotoxins but do not reduce their LAL activating properties. In this study, leucocytes showed no influence on endotoxin recovery in the LAL test. Further investigations still have to be carried out to gain insights into which plasma components are responsible for the neutralization of LPS in human blood. In addition to the existing recommendations for the sample preparation of blood for LAL measurements, such as heating and diluting, we could show in this study that the anticoagulation of blood samples has a major influence on the endotoxin recovery in the LAL assay. The output of this study clearly shows that the endotoxin recovery in heparin plasma is significantly higher than in serum. Blood donation, using citrate as anticoagulant, should be avoided for endotoxin measurements by the LAL assay. Since this study showed that endotoxin neutralization increases significantly with incubation time, plasma samples for LAL analysis should be processed rapidly. Overall, a lot of effort will have to be made to establish a standardized and reproducible procedure for endotoxin quantification in clinical blood samples by LAL-test. This would help to find out more quickly whether the predominant sepsis in patients is caused by Gram-negative infection. This could help to initiate a rapid and selective antibiotic treatment against Gram-negative bacteria. To conclude, the key findings of the present study are:

- Heparin increases the recovery and biological activity of endotoxins in human whole blood.
- Citrate decreases the recovery and biological activity of endotoxins in human blood.
- Physiological calcium and magnesium levels ensure maximum recovery of endotoxin in human blood.
- Human lipoproteins bind endotoxins, but do not influence their LAL activity and their monocyte stimulating properties.
- The endotoxin activity in blood decreases continuously during the first 6 h.
- Endotoxin neutralizing plasma components have a molecular mass smaller than albumin.
- Some of the endotoxin neutralizing plasma components are heparin-bound and have a molar mass between 12 and 13 kDa.

## Materials and methods

All experiments were conducted in accordance with the guidelines of the Declaration of the World Medical Association of Helsinki. The blood donation for this in vitro study was approved by the Ethics Committee of the Danube University Krems. Written consent to participate and approval for publication was obtained from each volunteer. Informed consent was obtained from all subjects.

### LAL assay

Endotoxin activity was measured using the kinetic chromogenic Limulus Amoebocyte Lysate (LAL) test (Charles River, Wilmington, USA). For the LAL analytic all samples were diluted 1:10 with pyrogen free water and incubated at 70 °C for 15 min, to denature plasma proteins and proteases which may interfere with endotoxin detection by the LAL assay^44,59^. 100 µL of each standard and sample was transferred into a 96-well flat bottom microplate in duplicates and incubated at 37 °C in a microplate photometer (Tecan sunrise, r-biopharm, Darmstadt, Germany) for 10 min. Then, 100 µL of Endosafe Endochrome-K LAL reagent, was quickly added. The photometer measured the increasing absorbance at 405 nm of each sample and standard. In the presence of endotoxin, the lysate will begin to cleave the chromogenic substrate, causing the solution to become yellow. The time required for the change is inversely proportional to the amount of endotoxin present. The concentration of unknown samples can then be calculated from a standard curve.

### Comparison of endotoxin recovery in aqueous solution, human plasma and serum

A spike recovery study of LPS in human plasma, serum and ringer solution was performed. For this purpose 5 ng/m LPS from *E. coli* O55:B5 (Sigma-Aldrich, St. Louis, Missouri, USA) were spiked in ringer solution (Fresenius Kabi, Bad Homburg, Germany), heparinized plasma (12 IU/mL), citrated plasma and serum and incubated on a roller mixer at 37 °C for 10 min. The LPS recovery was then determined by LAL test.

### Influence of different anticoagulants on endotoxin activity in plasma

Human serum from freshly drawn blood was spiked with increasing concentrations of different anticoagulants. The anticoagulants which were tested were high molecular weight heparin (Gilvasan, Vienna, Austria), fractionated low molecular weight heparin (Lovenox, EmraMed Arzneimittel GmbH, Germany), a selective inhibitor of activated factor X (Fondaparinux, Glaxo Group Ltd., UK) and citrate (gespag Bad Ischl pharmacy, Austria). For unfractionated heparin, fractionated heparin and fondaparanux, sera with 0, 0.1, 1, 2.5, 5 and 10 IU/mL were prepared. For testing the influence on endotoxin activity by citrate, serum samples were prepared with 0, 0.5, 1, 2, 3, 6, 9 and 12 mM citrate serum levels. For the LPS recovery study, 5 ng/mL LPS from *E. coli* was spiked into each serum sample and the LPS recovery was measured using the LAL test.

### Influence of heparin and citrate dose on the stimulating property of endotoxin on monocytes

Human serum from freshly drawn blood was spiked with increasing concentrations (0, 0.1, 0.5, 1, 2.5, and 5 IU/mL) of unfractionated heparin and increasing concentration of citrate (0, 1, 2, 3, 4, 6, 9 mM). After adding 10 ng/mL lipopolysaccharide from *E. coli* O55:B5 to the serum samples they were mixed with blood cells from the same donor in a ratio of 1:1 (v:v) and incubated on a roller mixer at 37 °C for 4 h. After incubation period sample were taken for quantifying pro-inflammatory cytokines (IL-1β, Il-6, IL-8 and TNF-α) using a magnetic Luminex assay from Bio-Rad Laboratories (Bio-Plex Pro Assays, Vienna, Austria).

### Preparation of lipoprotein-depleted and lipoprotein-enriched serum

Larger amounts of serum were prepared from citrated plasma (Red Cross, Linz, Austria). Calcium chloride was added to 4 L of citrated plasma until an ionized calcium level of 1.5 mM was reached. This was checked by means of an ionometer (electrolyte analyzer + 8, NOVA biomedical corporation, Waltham, USA). After 2 h incubation at room temperature the plasma was completely clotted. By centrifugation and filtration through a paper filter, the serum was separated from the clot. To obtain serum with physiological calcium and magnesium levels and to remove the citrate calcium complex from the serum, it was treated with a dialysis machine (multiFiltrate, Fresenius medical care, Bad Homburg, Germany). The multiFiltrate machine was prepared for CVVHD treatment. Therefore, a mulitFiltrate Kit CVVHD EmiC2 was used including the multiFiltrate cassette (arterial and venous tubing) and dialysate tubing system. The dialysate (multiBiC, Fresenius Medical Care, Bad Homburg, Germany) used for these trials contains 140 mM sodium, 1.5 mM calcium, 0.75 mM magnesium and 4 mM potassium. The tubing system including the EMiC2 filter was flushed with two liter of physiological saline before starting the experiment. The CVVHD treatment was carried out with a serum flow of 200 mL/min, a dialysate flow of 50 mL/min, without ultrafiltration for 4 h.

**Figure 18:**
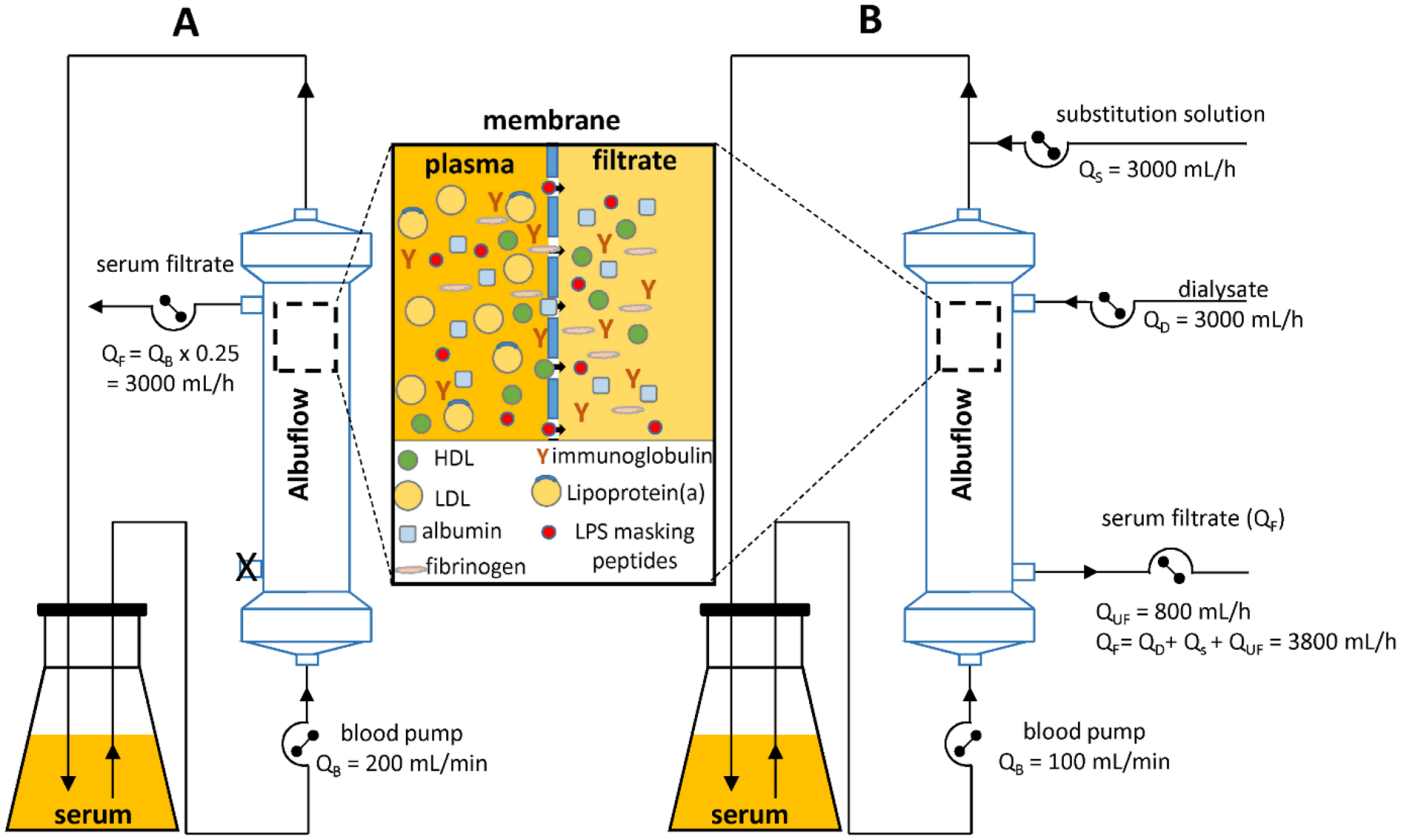
Schematic representation of the production of lipoprotein-enriched and lipoprotein-depleted serum. (**A**) Lipoprotein-depleted serum was obtained by filtration of serum through the Albuflow plasma filter, which has a molecular cut off of 250 kDa. (**B**) Lipoprotein-enriched serum was produced by haemodiafiltration with postdilution and ultrafiltraion. *Q*_*F*_ flow rate of serum filtrate, *Q*_*B*_ flow rate of the blood pump, *Q*_*S*_ flow rate of substitution solution (postdilution), *Q*_*UF*_ flow rate ultrafiltration, *Q*_*D*_ flow rate of dialysate.

Lipoprotein-depleted serum was obtained by using the plasma filter Albuflow (Fresenius Medical Care, Bad Homburg, Germany) which has an molecular cut off of about 250 kDa and a sieving coefficient of ≥ 60% for albumin. For this purpose, 2 L of serum were circulated through the plasma filter at a flow rate of 200 mL/min. On the secondary side of the filter, 200 mL filtrate was separated with a flow rate of 50 mL/min (Fig. 18A). The resulting lipoprotein-depleted serum filtrate was then divided into 10 mL aliquots and stored at − 30 °C. Lipoprotein-enriched serum was prepared by hemodiafiltration using the Albuflow filter (Fig. 18B). In hemodiafiltration, post-dilution with calcium-containing dialysate and ultrafiltration (800 mL/h) were performed. This led to a concentration of high molecular weight plasma components (lipoproteins, fibrinogen, immunoglobulins…) and to the washing out of plasma substances that can enter the Albuflow membrane. Hemodiafiltration with post-dilution was performed for 2 h to finally obtain 200 mL lipoprotein-enriched serum. Lipoprotein, albumin and total protein content of native, lipoprotein-enriched and lipoprotein-depleted serum were determined using the Cobas c311 analyzer from Roche (Basel, Switzerland) with according test reagents.

### Endotoxin recovery in lipoprotein-depleted and -enriched serum

5 ng/mL LPS from *E. coli* was spiked into native, lipoprotein-depleted and lipoprotein-enriched serum. Serum samples were incubated on a roller mixer at 37 °C for 1 h. After sampling, the LPS recovery was measured using the kinetic chromogenic Limulus Amoebocyte Lysate (LAL) test.

### Filtration of endotoxins in dependence of lipoprotein content

10 ng/mL LPS from *E. coli* was spiked into PBS (0.01 M), native serum, lipoprotein-depleted serum, lipoprotein-enriched serum and 4% (w/v) human serum albumin solution (Kedrion Biopharma, Barga, Italy). Each solution was filtered through a 0.22 µm and a 0.1 µm sterile filter from Merck (Burlington, USA), and centrifuged through Vivaspin 500 µL concentrators from Sartorius (Göttingen, Germany) with molecular cut offs of 100, 300 and 1000 kDa. In order to determine the sieving coefficient of LPS through the different filters, the LPS activity of the samples before and after filtration was determined using the LAL test.

### Filtration of endotoxin neutralizingn plasma compounds

Fresh serum from healthy donors was spiked with 3 µg/mL β2-microglobulin (Sigma-Aldrich, St.Louis, USA) and subsequently filtered through Vivaspin 500 µL concentrators from Sartorius (Göttingen, Germany) with molecular cut offs of 10, 30, 50, 100, 300 and 1000 kDa. In serum and the respective filtrate, the albumin and β2-MG concentrations were determined using Cobas c311 analyzer with the respective test kit. The sieving coefficient (S) in percent of albumin and β2-MG for each membrane was calculated using the following formula:

1 $$S\; \left[ \% \right] = \frac{{C_{serum} }}{{C_{filtrate} }} \times 100$$

where C_serum_ stands for serum and C_filtrate_ for filtrate concentration.

Additionally, the endotoxin recovery of 5 ng/mL (*E. coli*) in each filtrate was determined by LAL test.

### Change of endotoxin activity as a function of incubation time (reverse mode—kinetics)

Reverse endotoxin neutralization kinetics was prepared by spiking aliquots of a sample at different time points. The particular spiked and not spiked aliquoted were stored under equal conditions over time. The aliquot with the longest endotoxin incubation period was spiked first (24-h sample). Further aliquots with shorter incubation periods were spiked later in accordance with the respective incubation period. After spiking the time point 10 min aliquot, all samples were equivalently prepared and measured on the same assay. The endotoxin activity as a function of incubation time was studied in ringer solution, 4% (w/v) HSA solution, lipoprotein-enriched serum, fresh drawn serum and heparin plasma from healthy volunteers. Aliquots of each solution were made and incubated on a roller mixer at 37 °C. At certain times 5 ng/mL LPS form *E. coli* was spiked to the corresponding aliquots to obtain LPS incubation times of 10 min, 30 min, 1 h, 2 h, 3 h, 4 h, 6 h and 12 h. After incubation, all samples were immediately analyzed using the kinetic chromogenic LAL test.

### Endotoxin measurement of citrate anticoagulated plasma and serum

Kinetic endotoxin neutralization was also determined in citrated plasma and serum added with citrate before LAL measurement. When using citrated plasma, fresh blood from healthy donors was collected in citrate tubes. The citrate concentration in the centrifuged plasma was 18 mM. The citrated plasma was then mixed with 5 ng/mL LPS (*E. coli*) at different times to obtain different incubation times of LPS in citrated plasma (reverse mode). To determine whether the order of LPS addition (first LPS then citrate) has an influence on endotoxin activity, fresh serum was obtained from healthy donors. Reverse endotoxin neutralization kinetics was prepared by spiking aliquots of a sample at different time points. After the incubation period, 36 µL citrate solution (500 mM) was added per mL serum sample to obtain a citrate serum level of 18 mM. Prior to LAL measurement, the citrate-containing plasma and serum samples were diluted 1:10 with ringer solution instead of water to achieve an iCa level of 1 mM.

### Endotoxin activity in serum as a function of divalent cations

The ionized calcium (iCa) and magnesium (iMg) levels in blood are very precisely adjusted, as many biochemical processes (e.g. clotting cascade, complement system) require calcium or magnesium as cofactor. The normal range of iCa is between 1.15–1.35 mM and that of iMg between 0.75–1.0 mM.

Calcium- and magnesium-free serum was prepared using a cation exchanger. For this purpose, the respective serum was mixed in a ratio of 9:1 (serum:adsorbent) with the cation exchanger (Dowex 50WX8, Sigma-Aldrich, St. Louis, USA) and incubated for 1 h at room temperature on a roller mixer. The adsorbent exchanged calcium and magnesium from serum against sodium ions from the adsorbent surface.

To demonstrate the influence of divalent cations on endotoxin activity, the adsorbent treated serum and a 4% (w/v) HSA solution, diluted in PBS, and pyrogen-free water were spiked with increasing concentrations of calcium, magnesium and a combination of calcium and magnesium in a physiologically 2:1 ratio. The ionized calcium and magnesium content of the native and adsorbent treated serum samples were measured with an electrolyte analyzer (electrolyte analyzer + 8, NOVA Biomedical Corporation, Waltham, USA). The detection limit for iCa and iMg in serum is 0.1 mM, which is reached from a citrate serum level of 9 mM. After spiking 5 ng/mL LPS (*E. coli*) into the samples with different calcium and magnesium levels, LAL activity was measured immediately.

### Effect of endotoxin neutralization by plasma on Toll-like receptor activation

To determine whether the incubation period of LPS in plasma also has an influence on LPS activation of the Toll-like receptor 4 of monocytes, separate tests were performed. Fresh heparin blood was collected from healthy donors. Plasma was separated from blood cells by centrifugation (3000*g*, 10 min). As described above, plasma samples were prepared with different LPS incubation times (reverse mode). The plasma samples were then mixed with fresh blood cells from the same donor in a ratio of 1:1 (plasma volume:blood cell volume) and incubated for 4 h at 37 °C on a roller mixer. After the incubation period, plasma samples were frozen at − 80 °C for subsequent cytokine quantification (IL-1β, IL-6, IL-8 and TNF-α).

### Influence of leucocytes on endotoxin activity in blood

The endotoxin activity of 5 ng/mL LPS (*E.coli*) was compared between leucocyte-depleted blood and whole blood. As the endotoxin activity is influenced by heparin, additional measurements were performed with serum. Fresh heparin blood and serum from voluntary donors was collected. To deplete the leucocytes, the heparin blood was centrifuged (3000*g*, 10 min) and the buffy coat was carefully aspirated with a pipette. The plasma was then mixed with the leucocyte-depleted blood cells (− buffy coat) or with the whole blood cells (+ buffy coat) in a ratio of 1:1 (v:v). After adding 5 ng/mL LPS to the respective blood samples, the samples were incubated at 37 °C on a roller mixer. Samples for the measurement of endotoxin activity by LAL test were taken after 10 min and after 4 h. The same test approach was performed with serum instead of plasma. To remove heparin, the blood cells were washed 3 times with physiological saline solution and then mixed with the serum.

### Separation of heparin-binding plasma compounds

25 mL human serum from freshly drawn blood was pumped (0.5 mL/min) through a column filled with 5 mL heparin immobilized adsorbent (Heparin Sepharose 6 fast flow, GE Healthcare, Chicago, USA). To remove all non-heparin-binding substances from the adsorbent surface, the column was rinsed with 10 mL physiological saline solution. The heparin-bound plasma components were eluted from the column in two steps. Using 10 mL of 0.5 M NaCl solution, first those substances with a low to medium binding constant to heparin were eluted from the column. Substances strongly bound to heparin were finally eluted with 10 mL 2.2 M NaCl solution. The eluates were separated into 1 mL fractions using a fraction collector from (Bio-Rad, California, USA). Protein concentration of each fractions was analyzed with a Cobas c311 analyzer from Roche (Basel, Switzerland) with according test reagents. Fractions of each salt concentration were pooled and desalinated and concentrated with VIVASPIN 20 with a molecular cut off of 3 kDa (Sartorius, Göttingen, Germany). The concentrate of each fraction was washed once and finally diluted to a volume of 1.5 mL with ringer solution. Finally, 1:10 with ringer solution diluted serum and the two heparin-binding serum fractions (0.5, 2.2 M NaCl) were spiked with 5 ng/mL LPS (*E. coli*) and their endotoxin activity was determined by the LAL test.

### Gel electrophoreses

Native serum and the heparin-binding serum fractions were separate by SDS-PAGE and protein bands were visualized by staining with coomassie blue. A tricine 16% gel was used to monitor proteins from 1.7 to 40 kDa. Gels and the used running buffer (MES SDS buffer) were obtained from Thermo Fisher Scientific (Waltham, UK).

### Statistical analysis

All tests were carried out at least in triplicates. Calculations of mean and standard deviations were carried out using Microsoft Excel 2010 (Microsoft, WA, USA). All other statistical calculations were carried out with SigmaStat for Windows 2.03. The Kolmogorov–Smirnov Test was applied in order to check the data for normal distribution. Normal distributed data were compared using the *t* test. For data showing no normal distribution, the Mann–Whitney Rank Sum Test was used. p-values of ≤ 0.05 were considered as statistically significant.
